# Inhibition of Neuraminidase Inhibitor-Resistant Influenza Virus by DAS181, a Novel Sialidase Fusion Protein

**DOI:** 10.1371/journal.pone.0007838

**Published:** 2009-11-06

**Authors:** Gallen B. Triana-Baltzer, Larisa V. Gubareva, Alexander I. Klimov, David F. Wurtman, Ronald B. Moss, Maria Hedlund, Jeffrey L. Larson, Robert B. Belshe, Fang Fang

**Affiliations:** 1 NexBio, Inc., San Diego, California, United States of America; 2 Centers for Disease Control and Prevention, Atlanta, Georgia, United States of America; 3 Saint Louis University Medical Center, St. Louis, Missouri, United States of America; Singapore Immunology Network, Singapore

## Abstract

Antiviral drug resistance for influenza therapies remains a concern due to the high prevalence of H1N1 2009 seasonal influenza isolates which display H274Y associated oseltamivir-resistance. Furthermore, the emergence of novel H1N1 raises the potential that additional reassortments can occur, resulting in drug resistant virus. Thus, additional antiviral approaches are urgently needed. DAS181 (Fludase®), a sialidase fusion protein, has been shown to have inhibitory activity against a large number of seasonal influenza strains and a highly pathogenic avian influenza (HPAI) strain (H5N1). Here, we examine the *in vitro* activity of DAS181 against a panel of 2009 oseltamivir-resistant seasonal H1N1 clinical isolates. The activity of DAS181 against nine 2009, two 2007, and two 2004 clinical isolates of seasonal IFV H1N1 was examined using plaque number reduction assay on MDCK cells. DAS181 strongly inhibited all tested isolates. EC50 values remained constant against isolates from 2004, 2007, and 2009, suggesting that there was no change in DAS181 sensitivity over time. As expected, all 2007 and 2009 isolates were resistant to oseltamivir, consistent with the identification of the H274Y mutation in the NA gene of all these isolates. Interestingly, several of the 2007 and 2009 isolates also exhibited reduced sensitivity to zanamivir, and accompanying HA mutations near the sialic acid binding site were observed. DAS181 inhibits IFV that is resistant to NAIs. Thus, DAS181 may offer an alternative therapeutic option for seasonal or pandemic IFVs that become resistant to currently available antiviral drugs.

## Introduction

On the global scale, “classical” seasonal influenza results in 250,000–500,000 deaths and three to five million cases of severe illness annually [1]. Yearly vaccination is important for preventing influenza virus (IFV) infection. However, because of antigenic shift and antigenic drift, and the guesswork involved in predicting the dominant strains in future seasons, vaccines need to be updated every year and may not always be protective. Furthermore, production challenges for novel viruses or multi-strain epidemics threaten the supply of needed vaccine (e.g. current pandemic vaccine efforts reducing seasonal vaccine production). Finally, the host protective immune response may not be adequate in certain populations. Antiviral compounds are therefore needed for treating infected individuals, particularly during a severe or novel IFV outbreak. A well-recognized limitation of currently available antivirals is the risk of development of drug resistance. The emergence of drug-resistant IFV strains is a major public health concern in light of the burden of seasonal influenza and the ongoing pandemic of the 2009 A(H1N1) virus. The predicament of antiviral resistance is evident in the rapid establishment of viral resistance to M2 inhibitors (adamantanes) and the dramatic rise of oseltamivir-resistant seasonal H1N1 influenza in recent years [2]–[8].

While neuraminidase inhibitor (NAI)-resistance has been observed to occur via different molecular mechanisms [9], the dominating change conferring oseltamivir-resistance in the current seasonal IFV is a mutation in the neuraminidase (NA) gene, H274Y. This mutation has been also observed in oseltamivir-treated patients infected with the H5N1 HPAI, a troubling observation given the pandemic potential and extremely virulent nature of this IFV strain [10]–[12]. The frequency of isolates with the H274Y mutation has increased with each flu season, including in countries that do not regularly prescribe oseltamivir [2], [5]–[7]. According to CDC reports, the frequency of oseltamivir-resistance in seasonal isolates of H1N1 collected in USA grew from less than 0.5% in 2006–2007, to 13% in 2007–2008, to 99% in 2008–2009 (http://www.cdc.gov/flu/weekly/weeklyarchives2008-2009/weekly15.htm). Interestingly, this H274Y mutation was once believed to confer reduced viral fitness. However, because of the ease of transmission and significant pathogenicity in high risk patients, it is now concluded that the current oseltamivir-resistant H274Y mutant likely possesses the same degree of virulence as the wild-type strain [2], [6], [13]. Novel classes of anti-IFV compounds are needed, in particular to combat strains resistant to current drugs [14].

DAS181 (Fludase®) is a recombinant fusion protein composed of the catalytic domain of *Actinomyces viscosus* sialidase and the epithelial anchoring domain of human amphiregulin. DAS181 efficiently binds to respiratory epithelial cells and then removes cell-surface sialic acid residues [15], [16]. Sialic acid is the receptor which mediates IFV binding and entry into the host cell; therefore, removal of sialic acid by DAS181 potently inhibits IFV infection [15], [17]. By targeting the host cells rather than the virus, DAS181 may be less likely to induce drug resistance than virus-targeting compounds (e.g. adamantanes and NAIs). Long term DAS181 exposure to numerous cell lines and human primary cells does not cause cytotoxicity (Figures S1-2 and Methods S1). In numerous animal studies, as well as in ongoing phase 1 clinical trials, DAS181 is well-tolerated ([15] and unpublished data). In guinea pig and mouse asthma models, DAS181 does not cause airway hyperreactivity (unpublished data).

DAS181 has previously been reported to inhibit a large number of laboratory IFV strains and some clinical isolates both *in vitro* and *in vivo* [15], [18]. Here we report activity of DAS181 against a panel of seasonal IFV clinical isolates from recent seasons that are resistant to oseltamivir. A number of the 2009 seasonal IFV clinical isolates also exhibited changes in the HA that potentially reduced the virus' dependence on NA activity for virus release. Those clinical isolates exhibited decreased sensitivity in the MDCK cell assay to both NAIs, oseltamivir and zanamivir.

## Results

To evaluate activity of DAS181 against recent clinical isolates expected to be resistant to oseltamivir, we obtained nine seasonal IFV H1N1 isolates collected from patients at St. Louis University in the months of January and February of 2009. For comparison we obtained from CDC two pairs of seasonal IFV reference viruses: one pair is sensitive to both NAIs (2004 isolates) and the other pair is resistant to oseltamivir, due to the H274Y mutation in the NA (2007/08 season isolates, representative of two distinctive clades, “Hawaiian” and “Northern European”). The 2004 H1N1 clinical isolates were presumed to be oseltamivir sensitive, since no oseltamivir-resistance has been reported in community isolates prior to 2007. All isolates were tested for sensitivity to DAS181 using the plaque number reduction assay (PRA) with MDCK cells. The NAIs oseltamivir and zanamivir were also tested in tandem as controls. EC50s for each drug were calculated for each strain and compared to the 2004 strains in order to estimate fold resistance.

**Figure 1:**
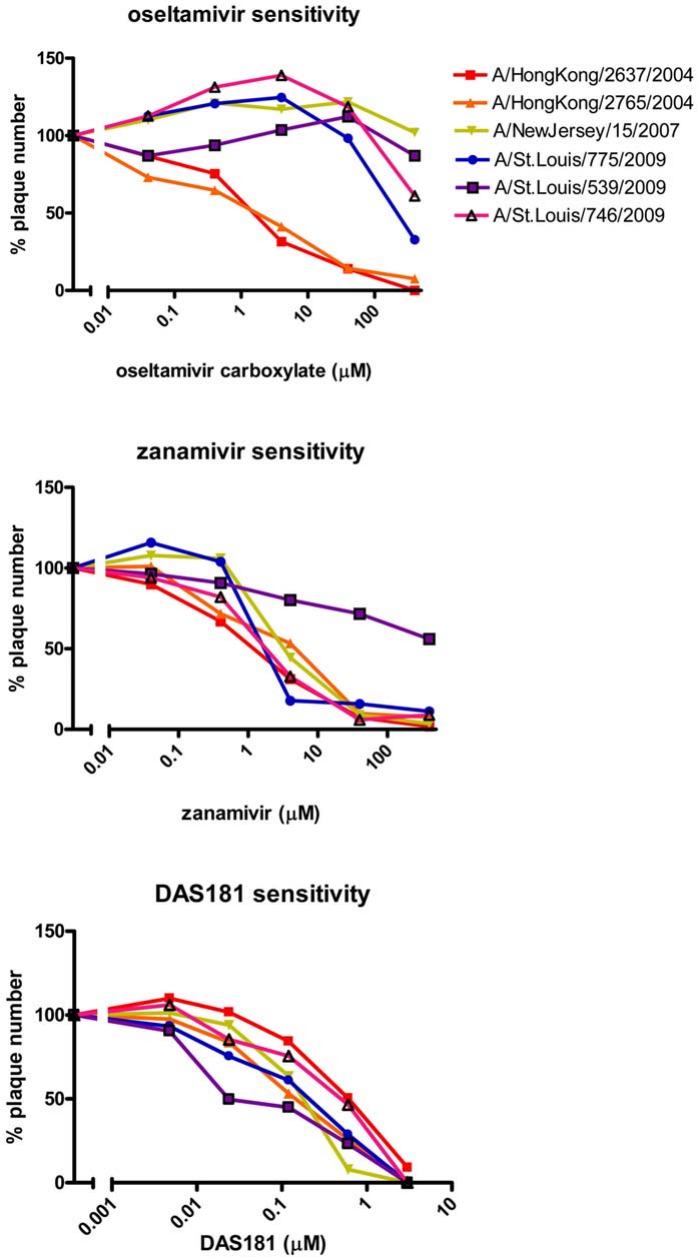
*In vitro* sensitivity of recent clinical isolates to oseltamivir, zanamivir and DAS181. Confluent MDCK cells were infected with clinical isolates of IFV A (H1N1) (from 2004, 2007, or 2009) and drug sensitivity was determined by plaque reduction assay (PRA). The number of viral plaques with each drug concentration was counted and plaque number was normalized against the untreated control. The graphs shown here represent a single PRA from a subset of all viruses tested (Table 1). The last two digits of each virus name indicate the year it was collected.

Dose response curves of the 2004, 2007, and 2009 isolates showed reduced sensitivity to oseltamivir and varied sensitivity to zanamivir in the 2009 isolates (Figure 1). Quantification revealed that EC50 values of oseltamivir against the 2007 and 2009 isolates were >100 times higher than that against the 2004 isolates (Table 1), indicating over 100-fold drug resistance for the 2007 and 2009 isolates in MDCK cells. Interestingly, EC50 values of zanamivir appeared to be elevated against 4 of the 9 isolates from 2009 (Table 1). All of the IFV clinical isolates were highly sensitive to DAS181 (Figure 1, Table 1). EC50 values of DAS181 were similar against each of the 2004, 2007, and 2009 isolates (Table 1). Of note, 3 of the 4 isolates from 2009 that appeared to have reduced sensitivity to both NAIs were approximately 10-fold more sensitive to DAS181 in MDCK cells (Table 1).

**Table 1:** *In Vitro* sensitivity of recent clinical isolates to NAIs and DAS181.

Virus (Year isolated in bold)	EC50 oseltamivir (uM)	EC50 zanamivir (uM)	EC50 DAS181 (uM)
A/HongKong/2637/**04**	3.27±1.32	1.82±0.46	0.49±0.06
A/HongKong/2765/**04**	2.92±1.00	4.17±0.53	0.26±0.13
A/NewJersey/15/**07**	>400	7.02±2.46	0.20±0.01
A/Hawaii/21/**07**	>285	45.03±22.53	0.29±0.02
A/St.Louis/539/**09**	>400	>332	0.020±0.001
A/St.Louis/746/**09**	>400	1.96±0.42	0.75±0.20
A/St.Louis/775/**09**	>320	1.20±0.36	0.25±0.06
A/St.Louis/758/**09**	>400	7.06±2.84	0.09±0.03
A/St.Louis/630/**09**	>400	62.10±34.84	0.14±0.02
A/St.Louis/764/**09**	>400	>273	0.030±0.002
A/St.Louis/690/**09**	>400	>182	0.51±0.14
A/St.Louis/790/**09**	>304	3.12±1.83	0.28±0.06
A/St.Louis/792/**09**	>400	>156	0.04±0.01

Values represent mean±SEM of triplicate plaque number reduction assay (PRA) experiments.

Upon sequencing the neuraminidase (NA) gene of the clinical isolates, we found that all of the tested 2007 and 2009 isolates, but not the 2004 isolates, contained the NA H274Y mutation known to confer oseltamivir resistance. No additional mutation in the NA gene was found that was specific to each of the isolates with apparently reduced zanamivir sensitivity. An N32S mutation was found in all but one zanamivir-sensitive 2009 isolate (A/St.Louis/758/09) and an I222V mutation was observed solely in two zanamivir-resistant 2009 isolates (Figure S3 and S4). We further characterized the NAI-resistance of the 2009 isolates by the NA activity inhibition (NI) assay, which measures the NA enzymatic activity in the presence of oseltamivir or zanamivir. In the NI assay, oseltamivir failed to effectively inhibit NA activity from each of the 2007 and 2009 isolates; however, all of these isolates were equally sensitive to zanamivir, including the 2009 isolates that carry the N32S and I222V mutations (Table 2). Thus, the NA activity of the 2009 isolates is still effectively inhibited by zanamivir, consistent with the lack of NA mutations specific to all the isolates that are less sensitive to zanamivir.

**Table 2:** Sensitivity of the 2009 clinical isolates to NAIs based on NA enzymatic activity.

Category	Virus	Oseltamivir IC50 (nM)	R/S	Zanamivir IC50 (nM)	R/S
**Reference virus**	A/Georgia/17/2006 WT	0.37	S	0.44	S
	A/Georgia/20/2006 H274Y	192.42	R	0.57	S
	A/New Jersey/15/2007	123.54	R	0.49	S
	A/Hawaii/21/2007	109.53	R	0.43	S
**2009 clinical isolate**	A/St.Louis/539/09	84.92	R	0.28	S
	A/St.Louis/746/09	89.93	R	0.3	S
	A/St.Louis/775/09	91.85	R	0.33	S
	A/St.Louis/758/09	88.41	R	0.34	S
	A/St.Louis/630/09	224.37	R	0.48	S
	A/St.Louis/764/09	102.82	R	0.3	S
	A/St.Louis/690/09	178.37	R	0.44	S
	A/St.Louis/790/09	87.82	R	0.29	S
	A/St.Louis/792/09	91.47	R	0.28	S

The 2006 and 2007 viruses (top 4) were included as reference strains.

S = Sensitive to drug. R = Resistant to drug.

**Figure 2:**
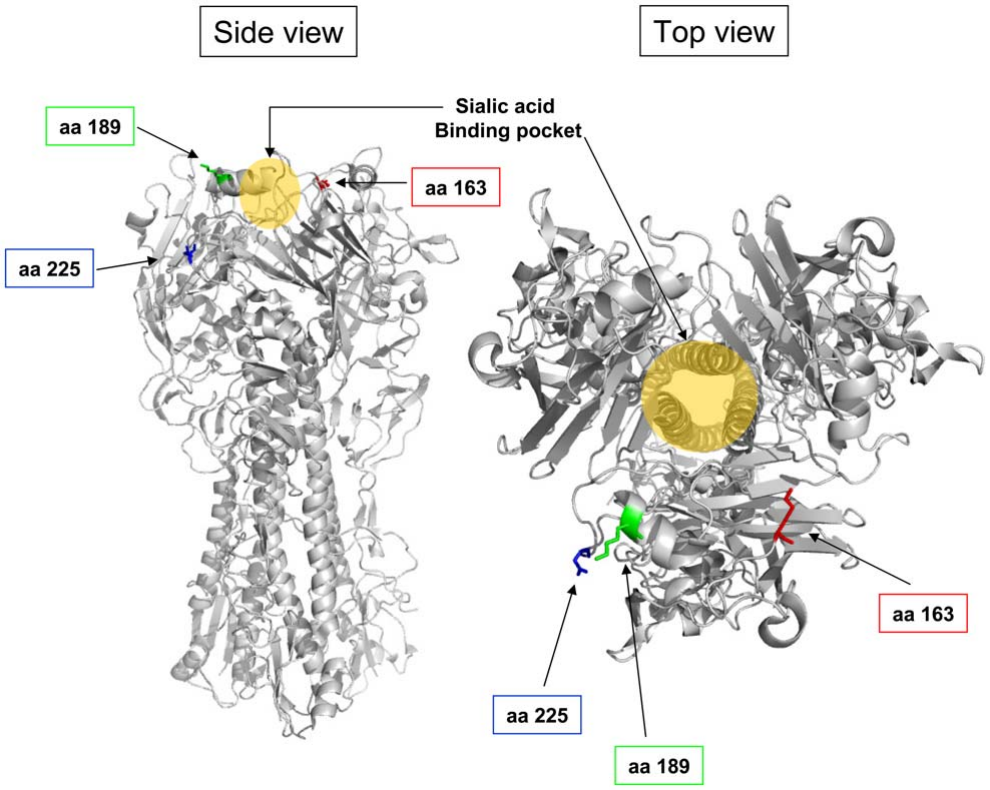
Location of hemagglutinin mutations specific to isolates with reduced zanamivir sensitivity. The amino acid positions for the mutations listed in Table 3 were located on the published crystal structure of an IFV A H1 trimer (1RU7). Amino acid 163 is shown in red, 189 in green, and 225 in blue. Left panel shows a side view of the HA trimer (with sialic acid binding site on top and membrane-proximal region on bottom) and right panel shows a top down view of the HA trimer. Approximate site of sialic acid binding is indicated with arrows and yellow shaded area.

To further characterize the observed reduction in zanamivir sensitivity exhibited by some of the isolates on the PRA, we also performed sequence analysis of the HA gene in all of the isolates. Mutations at one of two positions in the HA gene, N163 and D225, were observed only in the viral isolates with reduced zanamivir sensitivity (Table 3, full alignment Figure S5). Non-conserved mutations at these two positions, N163K/H and D225G, thus may be sufficient to cause reduced sensitivity to NAIs in MDCK cells. Although a zanamivir-sensitive isolate, A/St.Louis/758/09, carries a N163T mutation, this isolate also has a unique, nearby V189A mutation that might negate the effects of the N163T mutation such that the virus remains sensitive to zanamivir (Table 3). Sequence alignment with a published IFV A H1 crystal structure reveals that N163, D225, as well as V189 lie near the sialic acid binding pocket of HA (Figure 2). Therefore, the observed mutations might affect receptor binding affinity of the HA protein in MDCK cells.

**Table 3:** Hemagglutinin (HA1) mutations specific to isolates with reduced zanamivir sensitivity.

Virus	EC50 Zanamivir (µM)	Amino Acid at Position 163	Amino Acid at Position 189	Amino Acid at Position 225
A/HongKong/2637/**04**	1.82±0.46	N	V	D
A/HongKong/2765/**04**	4.17±0.53	N	V	D
A/NewJersey/15/**07**	7.02±2.46	N	V	D
A/Hawaii/21/**07**	45.03±22.53	**K**	V	D
A/St.Louis/539/**09**	>332	N	V	D**/G**
A/St.Louis/746/**09**	1.96±0.42	N	V	D
A/St.Louis/775/**09**	1.20±0.36	N	V	D
A/St.Louis/758/**09**	7.06±2.84	**T**	**A**	D
A/St.Louis/630/**09**	62.1±34.84	N	V	D**/G**
A/St.Louis/764/**09**	>273	**H**	V	D**/G**
A/St.Louis/690/**09**	>182	N/**T**	V	D
A/St.Louis/790/**09**	3.12±1.83	N	V	D
A/St.Louis/792/**09**	>156	N	V	D**/G**

Each 2009 isolate was sequenced directly following three passages in MDCK cells, without plaque purification. Sequence heterogeneities were observed at some locations and alternate amino acids at such locations are shown in the table. Amino acids different from reference strains are noted in bold. Sequence data noted with H3 numbering scheme, as previously described [42].

## Discussion

Two classes of antivirals against IFV are currently on the market, the adamantanes (amantadine and rimantadine), and the NAIs (oseltamivir and zanamivir). Antivirals play an important role in the treatment of severe seasonal and pandemic influenza, but their effectiveness is limited by the potential for IFV to develop drug resistance. Fortunately, the current pandemic IFV strain of swine origin (2009 A (H1N1)) is still largely sensitive to oseltamivir, although it is resistant to adamantanes [19]. However, given the presence of rampant oseltamivir resistance among the current seasonal H1N1 IFV strains, it could be just a matter of time before the pandemic H1N1 virus acquires resistance to oseltamivir [20]. Indeed, 28 cases of 2009 A (H1N1) resistance to oseltamivir have been reported as of October 2, 2009 (http://www.who.int/csr/don/2009_10_02/en/index.html). Thus development of novel therapeutics with unique mechanism of action is of great importance to public health.

In our analysis, all eleven 2007 and 2009 seasonal IFV clinical isolates (11/11) exhibited resistance to oseltamivir when tested in MDCK cells. The high frequency of observed oseltamivir resistance and H274Y mutation in our 2009 H1N1 isolates correlates with worldwide reports of resistance in this season (http://www.cdc.gov/flu/weekly/weeklyarchives2008-2009/weekly15.htm). Here we report that DAS181 is a potent inhibitor of the recent IFV clinical isolates. There is no reduction in DAS181 sensitivity amongst all the tested IFV clinical isolates from 2004, 2007 and 2009, demonstrating that the H274Y mutation does not reduce DAS181 sensitivity.

It has been well established that IFV develops resistance to NAIs through two mechanisms: one, mutations in NA that decrease binding affinity to NAI drugs, such as the H274Y mutation in the current seasonal IFV; two, mutations in HA which decrease virus receptor binding affinity, thereby reducing dependence on NA [9]. While NA mutations tend to make IFV resistant to one NAI drug, HA mutations have the potential to confer resistance to all NAI drugs. However, it is essential to recognize that NAI resistance caused by HA mutations is host-specific. Changes in HA that reduce the virus binding to receptors in one host (e.g., MDCK cells) may not be the same that produce such effect in another host (e.g., humans). Therefore, viruses that show “pan-NAI resistance” in MDCK cells could be fully susceptible to NAI in humans and vice versa [21]–[23]. HA mutations may play a role in reduced susceptibility to NAIs in drug patients, although direct experimental evidence is lacking at this time. In addition to resistance issues, HA mutations can change the tropism of viral binding (upper vs. lower respiratory tract binding) and the antigenicity of the virus.

When HA sequence analysis was performed on viruses recovered from oseltamivir treated children, an HA S262N mutation was found in 1 out of 50 cases, but NA mutations were detected in 18% of the cases [24]. Additional HA mutations, with or without accompanying NA mutations, have also been reported in other studies [21], [25]–[28]. In spite of numerous HA mutations reported in clinical samples, there is a lack of documentation on HA-mediated resistance to NAI drugs in seasonal IFV due to the lack of an acceptable phenotypic assay that can be used for surveillance purpose and resistance diagnosis [9]. The commonly used MDCK cell PRA is considered unreliable for detecting NAI resistance in humans because it is prone to give false positive and false negative results [9]. The well differentiated human airway epithelium culture (HAE) is an experimental model system that better mimics the human airway, but it is cumbersome to grow and IFV clinical isolates tend to grow poorly in HAE cultures. Thus, the best option to identify HA mutations that may potentially give rise to NAI resistance may be to perform sequence analysis of the HA gene to specifically look for mutations at or near sialic acid binding site [9].

In the 2007 and 2009 clinical isolates that appear to have decreased sensitivity to zanamivir based on PRA, we have identified mutations involving two amino acid residues in the HA gene that are proximate to the sialic acid binding site, N163 and D225. Mutation of N163 may be highly relevant for NAI-resistance because glycosylation of asparagine (N) near the sialic binding site directly impacts the HA receptor binding affinity [29], [30]. Mishin et al. reported that introduction of an N163G mutation made a recombinant laboratory IFV strain more sensitive to NAIs in MDCK cells [31]. However in our analysis, the N163H/K/T mutation was primarily observed in isolates with reduced zanamivir sensitivity, which seems to indicate an opposite effect of mutation involving N163. This contradiction may be due to differences in the HA backbone sequence between the isolates tested here and the virus in Mishin et al. It also reflects the complexity of interpreting HA mutations. Nevertheless, these results together suggest significance of N163 residue in NAI sensitivity in MDCK cells.

Examination of the HA sequence of *pandemic* 2009 IFV finds that nearly all isolates have a lysine (K) at amino acid 163, yet exhibit normal zanamivir sensitivity. However, the framework sequence of the HA in the pandemic 2009 IFV is radically different from that of seasonal H1N1 IFV, and thus inferences about a single point mutation amidst the sea of other changes is tenuous at best. For *seasonal* influenza H1N1 an alignment of 2009 sequences prior to the swine flu outbreak (01/2009 to 03/2009) reveals that 85/89 have an N and 2/89 have K/H/T at amino acid 163, indicating that the N163K/H/T mutation is rare, but does exist at some low level in seasonal influenza (Influenza Virus Resources, [32]). Further, most testing of NAI sensitivity currently occurs with the NI assay, which would miss NAI-resistance due to HA mutations, and limited prescription of zanamivir might reduce the chance of observing clinical zanamivir resistance. Hence, N163K/H/T-mediated reduction in NAI sensitivity in humans is unlikely to have been observed as yet.

An NA mutation (I222V) was observed in two of the zanamivir-resistant 2009 seasonal IFV strains, in addition to the well described H274Y mutation. Interestingly this mutation has recently been observed, in tandem with the H274Y mutation, in 2 patients with pandemic 2009 IFV. Drug sensitivity analysis was not performed on these viral isolates, therefore the significance of the I222V mutation in patients is unclear [33]. Previous *in vitro* selection studies have indicated that the I222V mutation exacerbates oseltamivir- and peramivir-resistance caused by H274Y, but has only modest affect on zanamivir sensitivity [34], [35].

Broad-spectrum resistance to NAIs caused by a combination of HA and NA mutations has been previously reported when laboratory IFV strains were subjected to i*n vitro* passages in the presence of oseltamivir [36] or peramivir [37]. In the first case, a combination of A28T(HA) and R292K(NA) mutations resulted in 3230-fold and 60-fold resistance by A/Victoria/3/75 (H3N2) to oseltamivir and zanamivir, respectively [36]. In the second case, B/Yamagata/16/88 acquired 100 to 700-fold resistance to oseltamivir, zanamivir, and peramivir due to a H274Y mutation in NA in combination with six HA mutations (G141E, D195N, T197N, T139N, R162M, and Y319H) [37]. Similarly in our study, the 2007 and 2009 isolates that are resistant to oseltamivir and have reduced sensitivity to zanamivir carry mutations in both NA (H274Y) and HA (N163K/T/H and D225G), but the observed HA mutations have not been reported previously in drug-resistant IFV strains. The N163 and D225 amino acids are highly conserved amongst published H1N1 IFV strains (Figure S6), further indicating their potential importance in viral infection. Further elucidation in *in vivo* models is required to confirm the clinical significance of these mutations. Finally, an IFV A/Victoria/3/75 passaged with peramivir and found to be strongly pan-NAI resistant (oseltamivir, zanamivir, peramivir) *in vitro* [38], correlating with a single HA mutation (K186E), has been subsequently shown to be highly sensitive to DAS181 in MDCK cells and mice (unpublished data).

Distinct from the adamantanes and NAIs, DAS181 targets the host cells, not the virus. It functions by removing sialic acid receptors for IFV and thus rendering the host cells unable to be infected by IFV. Given the unique mechanism of action of DAS181, it is not unexpected that IFV strains that are resistant to antivirals remain sensitive to DAS181. Our results suggest that DAS181 could be an effective treatment for NAI-resistant IFV, whether the drug resistance is due to mutations in NA or HA. We have previously demonstrated that DAS181 potently inhibits the current pandemic H1N1 virus of swine origin *in vitro* and *in vivo* [39]. Thus, DAS181 may offer a potential therapeutic option for pandemic 2009 A(H1N1) virus resistant to NAI drugs.

## Materials and Methods

### Cells and Viruses

Madin Darby Canine Kidney (MDCK) cells were obtained from American Type Culture Collection (Manassas, VA). The 2004 and 2007 seasonal IFV clinical isolates were obtained from Alexander Klimov, Centers for Disease Control and Prevention. The 2009 clinical isolates of seasonal IFV collected in the months of February and March 2009 at Saint Louis University Hospital, (A/St.Louis/XXX/09) were generously provided by Robert Belshe, School of Medicine, Saint Louis University. Patient information was confidential and no additional medical or personal information was collected.

### Viral Titer Determination by Plaque Assay

All 2009 clinical isolates were amplified for 3 passages on MDCK cells after collection from human subjects before use in this study. All viruses used here were initially quantified on MDCK cells to determine infectious titer (plaque forming units per mL, pfu/ml). In brief 6x10-fold serial dilution was performed on the viral samples followed by 1 hr binding at 37°C on confluent MDCK cells in 6-well plate format. After washing off unbound virus with PBS, the cells were overlaid with 1∶1 Noble Agar (1.8%) and 2x DME-F12 (supplemented with Glutamax (Invitrogen, Carlsbad, CA), ITS (Invitrogen), and 3 µg/ml acetylated trypsin (Sigma, St. Louis, MO)). After allowing agar to solidify, the plates were incubated for ∼60 hrs at 37°C before fixing with crystal violet and counting plaque number at each dilution.

### Antiviral Compounds

Zanamivir (Relenza) was removed from Rotadisk blister packs (each containing 5 mg of active zanamivir and 20 mg of lactose as excipient) and reconstituted in deionized water with final zanamivir concentration of 10 mM. Tartrate salt of oseltamivir carboxylate (active form of Tamiflu) was generously provided by F.Hoffmann-La Roche (Basel, Switzerland) and reconstituted in deionized water such that the final oseltamivir carboxylate concentration was 10 mM. Reconstituted NAIs were stored at −20°C until use. Purified DAS181 was supplied in 1.7 mM acetate buffer, pH 5 and stored at −80°C until use.

### Drug Sensitivity Testing with Seasonal IFV Strains

The clinical isolates of seasonal IFV were tested for DAS181 sensitivity simultaneous with oseltamivir and zanamivir sensitivity using the plaque number reduction assay on MDCK cells. This protocol is modified from previous publication [40]. In short 150 pfu of virus are applied to each well of confluent MDCK cells in 6-well plate format. After binding for 1.5 hrs, unbound virus is washed off with PBS and the plates are overlaid with 1∶1 Noble Agar and DME-F12 as in the plaque assay. 2x DAS181, oseltamivir, or zanamivir concentrations are included in the 2xDME-F12, so as to achieve 1x final concentration in the agar/media mix. NAIs were tested at concentrations from.04 to 400 µM, while DAS181 was tested at concentrations from.0048 to 3 µM since no plaques remained with 3 µM DAS181. After allowing agar to cool the plates were incubated, stained, and counted as in the plaque assay. Data was graphed as plaque number per drug concentration, normalized to no drug and expressed as percent plaques remaining. EC50s were calculated as the concentration of drug reducing plaque number to 50% of no drug control. All viruses were tested in triplicate against each drug and values are represented as mean±SEM. All data was graphed with Prism 4.02 software.

### Genotyping

All 2004, 2007, and 2009 clinical isolates tested here were sequenced to determine the HA and NA genotypes. In brief, RNA was purified from 50 uL of viral sample using using MagMAX-96™ Viral RNA isolation kit (Applied Biosystems). The RNA was purified according to manufacturer's instructions and eluted in 50 µL elution buffer. PCR was performed on the RNA with primers to amplify the entire HA and NA genes (designed based on alignment of several H1N1 viruses). Upon confirming amplification of correct size band the PCR product was purified with PCR purification kit (Qiagen, Valencia, CA) and sequenced using the same forward and reverse primers. Oligonucleotide sequences were aligned (comparing all strains or only all 2009 strains) using ClustalW2 software (http://www.ebi.ac.uk/Tools/clustalw2/index.html) and sequence data noted with H3 or N2 numbering scheme, as previously described [41], [42]. Alignments of NA sequences are shown in Figure S3 and S4. Alignments of HA sequences are shown in Figure S5 and S6. Positions of mutated amino acids within crystal structure of HA trimer were determined by comparison to a published IFV A/PuertoRico/8/34 H1 trimer (1RU7) using PyMOL Software [43]. HA and NA nucleotide and amino acid sequences for the A/St.Louis/XXX/2009 isolates have been submitted in Genbank; Accession numbers for HA sequences: GQ994954, GQ994955, GQ994956, GQ994957, GQ994958, GQ994959, GQ994960, GQ994961, GQ994962. Accession numbers for NA sequences: GQ994963, GQ994964, GQ994965, GQ994966, GQ994967, GQ994968, GQ994969, GQ994970, GQ994971.

### NI Assay

The chemiluminescent neuraminidase activity inhibition (NI) assay was conducted using a commercially available kit, NA-Star (Applied Biosystems, Foster City, CA) as previously described [7]. Oseltamivir carboxylate and zanamivir were used at 10 half-log dilutions (0.03 to 1000 nM). Values shown represent mean of triplicate analysis.

## Supporting Information

Methods S1 Materials and methods for Figures S1 and S2.
(0.04 MB DOC)

Figure S1 Effect of DAS181 on cell line proliferation. To determine whether DAS181 affected cell proliferation, the growth of several immortalized cell lines were monitored in the presence and absence of 17 µM DAS181. A549 (A), MDCK (B), CACO-2 (C), or BEAS-2B (D) cell lines were first plated at subconfluent density. After 24 hrs the growth media was replaced with media containing DAS181, PBS, or cell culture medium alone and placed back at 37°C to incubate for 10 days. Relative cell numbers were determined daily by crystal violet staining. Values represent mean±SD of six replicates. DAS181 or PBS treatments were not significantly different from cell culture medium alone for any of the cell lines, as determined by ANOVA with Bonferroni post-test.
(0.06 MB PPT)

Figure S2 Cytotoxic effect of DAS181 on primary human renal epithelial cells. Human renal proximal tubule cells and cortical epithelial cells were exposed to various concentrations of DAS181 for 24 hrs and monitored for cell viability over 3 days (A–F) or exposed to DAS181 for 72 hrs and immediately assayed for cell viability (G–H). In all cases some cells were also exposed to PBS (vehicle control), or 0.2% thimerosol or 1 mM cadmium chloride (CdCl2) as positive controls for cell death. Cell viability was assessed by MTS assay. In all cases, treatment with either thimerosol or CdCl2 resulted in significant reduction in cell viability, however with minor exception, all DAS181 treatment levels/regimens were not significantly different from PBS. Values represent mean±SEM of triplicate samples. * = P<0.05, *** = P<0.001; significantly different from PBS as determined by ANOVA with Bonferroni post-test.
(0.37 MB DOC)

Figure S3 NA alignment of all 2009 isolates tested here. Primers designed to clone the entire NA gene were used to also sequence the NA gene. Data was obtained for the entire NA gene except the region corresponding to the first 27 and final 15 amino acids. Sequences were aligned with Clustal W2 software. Sequence data noted with N2 numbering scheme, as previously described [41]. Highlighted residues correspond to: Red = N32, Green = I222, Blue = H274. * = identical amino acid, :  = highly similar amino acid, .  = moderately similar amino acid. Accession numbers for NA sequences aligned here: A/St.Louis/790/2009 = GQ994970 A/St.Louis/764/2009 = GQ994968 A/St.Louis/630/2009 = GQ994964 A/St.Louis/792/2009 = GQ994971 A/St.Louis/775/2009 = GQ994969 A/St.Louis/690/2009 = GQ994965 A/St.Louis/539/2009 = GQ994963 A/St.Louis/758/2009 = GQ994967 A/St.Louis/746/2009 = GQ994966
(0.04 MB DOC)

Figure S4 NA alignment of recently published H1N1 IFV isolates. Published HA sequences for several 2007/2008 isolates were aligned with Clustal W2 software to determine the conservation of amino acid choice at select regions identified as mutations in Figure S1. Sequence data noted with N2 numbering scheme, as previously described [41]. Highlighted residues correspond to: Red = N32, Green = I222, Blue = H274. * = identical amino acid, :  = highly similar amino acid, .  = moderately similar amino acid. Accession numbers for NA sequences aligned here: A/Kentucky/UR06-0369/2007 = CY037665 A/Texas/UR06-0422/2007 = CY037441 A/Ohio/UR06-0493/2007 = CY037657 A/NewJersey/15/2007 = EU516148 A/Perth/33/2008 = FJ743472 A/Kentucky/UR07-0061/2008 = CY037697 A/Washington/AF06/2007 = CY037329 A/Florida/UR07-0022/2008 = CY037681 A/Hawaii/21/2007 = EU516112 A/Japan/AF07/2008 = CY037337 A/Cambodia/21/2007 = FJ743470 A/Tennessee/UR06-0106/2007 = CY037785 A/Vermont/UR06-0513/2007 = CY037465
(0.05 MB DOC)

Figure S5 HA alignment of all 2009 isolates tested here. Primers designed to clone the entire HA gene were used to also sequence the HA gene. Data was obtained for the entire HA gene except the region corresponding to the first 25–31 and final 12 amino acids. Sequences were aligned with Clustal W2 software. Sequence data noted with H3 numbering scheme, as previously described [42]. Highlighted residues correspond to: Red = N163, Green = G/V189, Blue = D225. * = identical amino acid, :  = highly similar amino acid, .  = moderately similar amino acid. Poor data was obtained for isolate 690 in region of first 100 amino acids so this sequence is omitted from alignment comparison here. Accession numbers for HA sequences aligned here: A/St.Louis/790/2009 = GQ994961 A/St.Louis/764/2009 = GQ994959 A/St.Louis/630/2009 = GQ994955 A/St.Louis/792/2009 = GQ994962 A/St.Louis/775/2009 = GQ994960 A/St.Louis/690/2009 = GQ994956 A/St.Louis/539/2009 = GQ994954 A/St.Louis/758/2009 = GQ994958 A/St.Louis/746/2009 = GQ994957
(0.04 MB DOC)

Figure S6 HA alignment of recently published H1N1 IFV isolates. Published HA sequences for several 2007/2008 isolates were aligned with Clustal W2 software to determine the conservation of amino acid choice at select regions identified as mutations in Figure S3. Sequence data noted with H3 numbering scheme, as previously described [42]. Highlighted residues correspond to: Red = N163, Green = G/V189, Blue = D225. * = identical amino acid, :  = highly similar amino acid, .  = moderately similar amino acid. Accession numbers for HA sequences aligned here: A/Kentucky/UR06-0369/2007 = CY037663 A/Texas/UR06-0422/2007 = CY037439 A/Ohio/UR06-0493/2007 = CY037655 A/NewJersey/15/2007 = EU516083 A/Perth/33/2008 = FJ743473 A/Kentucky/UR07-0061/2008 = CY037695 A/Washington/AF06/2007 = CY037327 A/Florida/UR07-0022/2008 = CY037679 A/Hawaii/21/2007 = EU516080 A/Japan/AF07/2008 = CY037335 A/Cambodia/21/2007 = FJ743471 A/Tennessee/UR06-0106/2007 = CY037783 A/Vermont/UR06-0513/2007 = CY037463 A/Charlottesville/31/95 = AF398878
(0.05 MB DOC)
