# Supplementary material for: Inhibition of Neuraminidase Inhibitor-Resistant Influenza Virus by DAS181, a Novel Sialidase Fusion Protein
Source: PLoS One. 2009 Nov 6;4(11):e7838. doi: 10.1371/journal.pone.0007838 (PMC2770896; doi:10.1371/journal.pone.0007838)
Supplement: Methods S1 — Materials and methods for Figures S1 and S2. (0.04 MB DOC) [file pone.0007838.s001.doc]

**Materials and Methods for Figures S1 and S2**

*Cells*

A549 (human alveolar basal epithelial) cells, MDCK (Madin Darby canine kidney) cells, and CAC0-2 (human colorectal epithelial) cells (ATCC, Manassas, VA) were grown in DMEM supplemented with 10% fetal bovine serum, 1x Glutamax (Invitrogen, Carlsbad, CA), and 1x Antibiotic/Antimycotic solution (Sigma, St Louis, MO) at 37ºC in a humidified atmosphere of 5% CO_2_. BEAS-2B (human bronchial epithelial) cells (ATCC) were grown in DMEM-F12 supplemented with 10% FBS, 1x Glutamax, and 1x Antibiotic/Antimycotic solution, and 1X non-essential amino acids (Invitrogen) at 37ºC in a humidified atmosphere of 5% CO_2_.

Primary cryopreserved human renal cells (Proximal Tubule Epethelial and Cortical Epethelial cells) were thawed, plated, and propagated according to Cambrex’s recommended protocol. Both renal cell lines were thawed and plated at a seeding density of 2500 cells/cm^2^ with Renal Epethelial Basal Medium (REBM) supplemented with Cambrex REGM SingleQuots® (Cambrex CC-4127); which includes recombinant human epidermal growth factor, insulin, GA-1000, 0.5% Fetal Bovine Serum, hydrocortisone, transferrin, epinephrine, and triiodothyronine (T3). The cells were allowed to adhere and equilibrate for 24 hours at 37°C with 5% CO_2_. Thereafter, media was changed at least every other day until the cells reached 60 to 70% confluency, taking care to ensure that they did not become fully confluent. All the renal cells were further subcultured and seeded into 10 tissue culture-coated 24 well plates per cell line at a seeding density of 2.5x10^3^ cells/cm^2^. Once the cells reached approximately 70% confluency the 24 well plates were used in cytotoxicity experiments using the REBM supplemented with the REGM SingleQuots®.

*Cell Growth Curves*

MDCK, A549 and CACO-2, were seeded into 96-well plates at 2.0 x 10^3^ cells/well. BEAS-2B cells were seeded into 96-well plates at 4.0 x 10^3^ cells/well. The cells were allowed to adhere to the plate overnight, and at 24 hrs post-plating the growth medium was exchanged with either fresh growth medium, growth medium with 0.8 mg/mL (17 μM) DAS181, or growth medium with vehicle control (PBS). Cells were then incubated at 37°C in a humidified atmosphere of 5% CO^2^. At various time points over a period of 10 days, the relative number of cells per well was determined by crystal violet uptake. The medium was aspirated and replaced with 100 μL/well 0.5% crystal violet in 20% methanol for five minutes at room temperature. The plates were then washed with water, and air dried. Bound crystal violet was then extracted with 70% ethanol and absorbance (570 nm) in the ethanol solutions was measured with a microplate reader. Absorbance values were plotted against time (hours). Since crystal violet stains living cells the absorbance readings are proportional to the relative number of cells. Each PBS and DAS181 growth curve was compared to the media alone growth curve, via ANOVA with Bonferroni post-test, to determine if there was a statistically significant change in cell proliferation.

*Primary cell cytotoxicity experiments*

On Day 0 the cell media supernatants were initially collected for baseline controls, before adding fresh media supplemented with either PBS, DAS181 (indicated concentrations), or thimerosal (0.2%) or CdCl^2^ (1mM) to serve as positive controls for cytotoxicity. All treatment groups were done in triplicate. Treatments were left on for either 24 hrs or 72 hrs. Subsequently, for the 24 hr exposure conditions fresh media without test article was added to all the wells every 24 hours on days 1-3 (24-72 hours post-treatment); a subset of the wells were used for MTS assay each day. For the 72 hr exposure conditions the media was only changed at 72 hrs, immediately prior to MTS assay. Renal cell media interfered with Lactate Dehydrogenase (LDH) and Alkaline Phosphatase (ALP) assays so these were not performed.

*MTS Assay*

At each endpoint (24, 48, or 72 hrs) the old media was first replaced with fresh warm media (500uL/well) and the samples were then tested for cell viability using the CellTiter 96® Aqueous Non-Radioactive Cell Proliferation Assay (MTS) kit (Promega, Madison, WI) [1]. 100μL of the MTS substrate was added to each well and the plate was allowed to incubate at 37°C for 1 to 4 hours until the samples gave a reading in the linear range suggested by Promega. 100μL of each sample was then added to a 96 well plate and read for absorbance at 490 nm. A 1:5 dilution of MTS solution in growth media alone was used as the background. For data analysis, background values were subtracted from all sample values. Values were then normalized using the vehicle control as 100%. Each sample was compared to the vehicle control, via ANOVA with Bonferroni post-test, to determine if there was a statistically significant change in cell viability.

Reference List

1. Mosmann T (1983) Rapid colorimetric assay for cellular growth and survival: application to proliferation and cytotoxicity assays. J Immunol Methods 65: 55-63.
