# Supplementary figures and images for: Inhibition of Neuraminidase Inhibitor-Resistant Influenza Virus by DAS181, a Novel Sialidase Fusion Protein
Source: PLoS One. 2009 Nov 6;4(11):e7838. doi: 10.1371/journal.pone.0007838 (PMC2770896; doi:10.1371/journal.pone.0007838)

## Slide 1
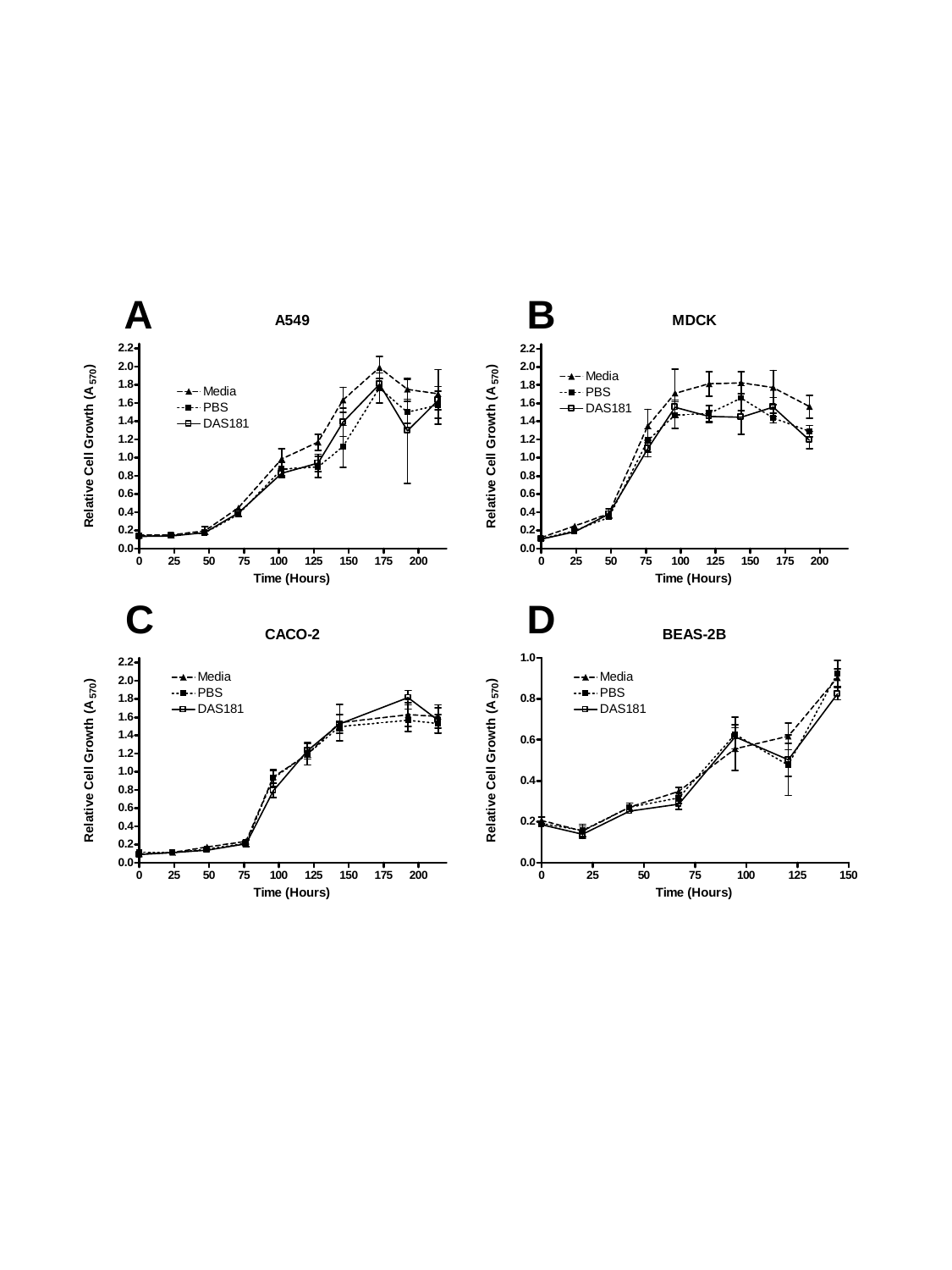

Supplement: Figure S1 — Effect of DAS181 on cell line proliferation. To determine whether DAS181 affected cell proliferation, the growth of several immortalized cell lines were monitored in the presence and absence of 17 µM DAS181. A549 (A), MDCK (B), CACO-2 (C), or BEAS-2B (D) cell lines were first plated at subconfluent density. After 24 hrs the growth media was replaced with media containing DAS181, PBS, or cell culture medium alone and placed back at 37°C to incubate for 10 days. Relative cell numbers were determined daily by crystal violet staining. Values represent mean±SD of six replicates. DAS181 or PBS treatments were not significantly different from cell culture medium alone for any of the cell lines, as determined by ANOVA with Bonferroni post-test. (0.06 MB PPT) [file pone.0007838.s002.ppt]

## Slide 1
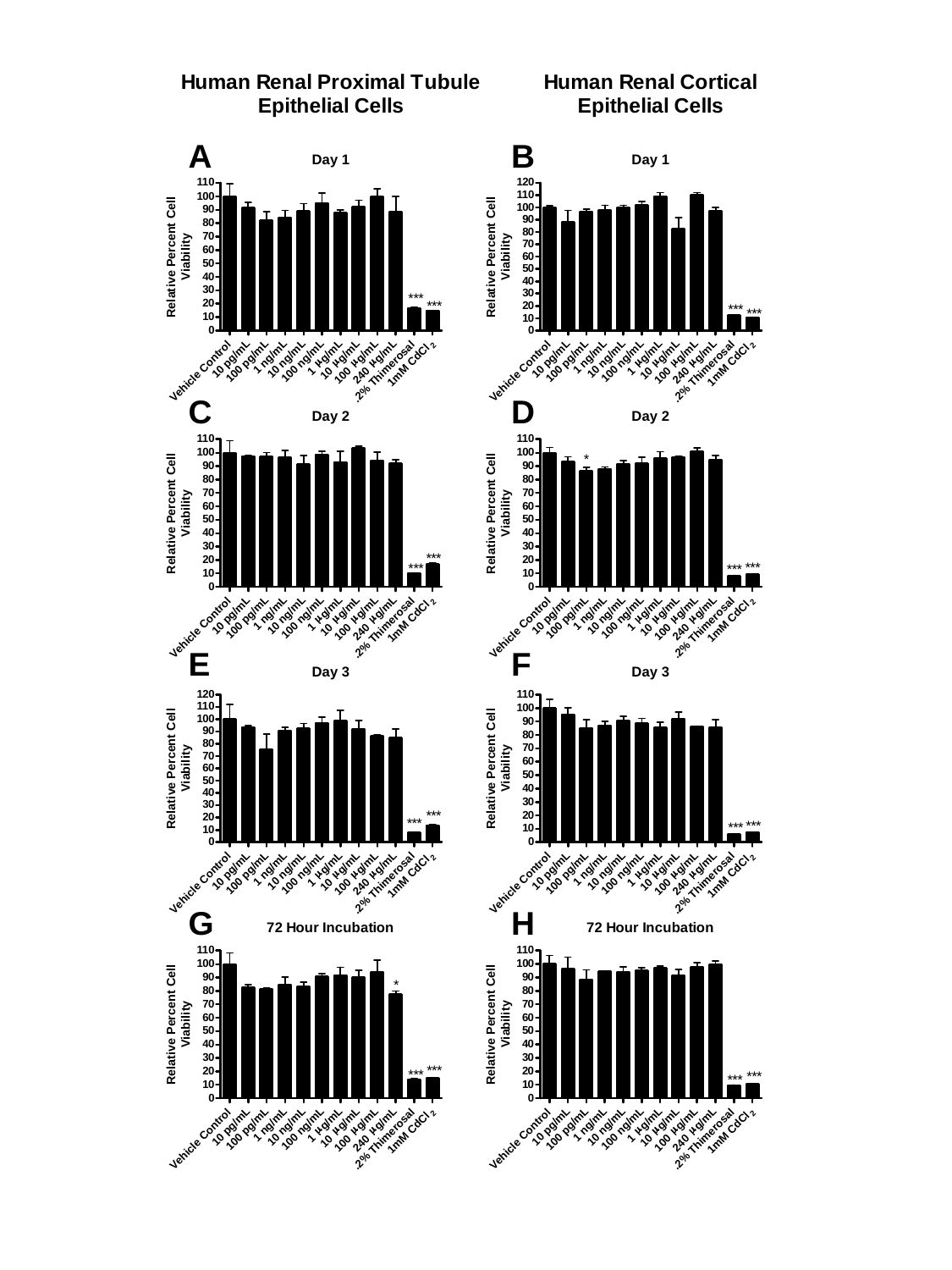

Supplement: Figure S2 — Cytotoxic effect of DAS181 on primary human renal epithelial cells. Human renal proximal tubule cells and cortical epithelial cells were exposed to various concentrations of DAS181 for 24 hrs and monitored for cell viability over 3 days (A–F) or exposed to DAS181 for 72 hrs and immediately assayed for cell viability (G–H). In all cases some cells were also exposed to PBS (vehicle control), or 0.2% thimerosol or 1 mM cadmium chloride (CdCl2) as positive controls for cell death. Cell viability was assessed by MTS assay. In all cases, treatment with either thimerosol or CdCl2 resulted in significant reduction in cell viability, however with minor exception, all DAS181 treatment levels/regimens were not significantly different from PBS. Values represent mean±SEM of triplicate samples. * = P<0.05, *** = P<0.001; significantly different from PBS as determined by ANOVA with Bonferroni post-test. (0.37 MB DOC) [file pone.0007838.s003.ppt]
