# Supplementary material for: Inhibition of Neuraminidase Inhibitor-Resistant Influenza Virus by DAS181, a Novel Sialidase Fusion Protein
Source: PLoS One. 2009 Nov 6;4(11):e7838. doi: 10.1371/journal.pone.0007838 (PMC2770896; doi:10.1371/journal.pone.0007838)
Supplement: Figure S3 — NA alignment of all 2009 isolates tested here. Primers designed to clone the entire NA gene were used to also sequence the NA gene. Data was obtained for the entire NA gene except the region corresponding to the first 27 and final 15 amino acids. Sequences were aligned with Clustal W2 software. Sequence data noted with N2 numbering scheme, as previously described [41]. Highlighted residues correspond to: Red = N32, Green = I222, Blue = H274. * = identical amino acid, : = highly similar amino acid, . = moderately similar amino acid. Accession numbers for NA sequences aligned here: A/St.Louis/790/2009 = GQ994970 A/St.Louis/764/2009 = GQ994968 A/St.Louis/630/2009 = GQ994964 A/St.Louis/792/2009 = GQ994971 A/St.Louis/775/2009 = GQ994969 A/St.Louis/690/2009 = GQ994965 A/St.Louis/539/2009 = GQ994963 A/St.Louis/758/2009 = GQ994967 A/St.Louis/746/2009 = GQ994966 (0.04 MB DOC) [file pone.0007838.s004.doc]

A/St.Louis/790/09 -----CCNR**S**IISIWASHSIQTGSQNNTGICNQRIITYENSTWVNHTYVNINNTNVVAGE 77

A/St.Louis/764/09 -----CCNR**S**IISIWASHSIQTGSQNNTGICNQRIITYENSTWVNHTYVNINNTNVVAGE 77

A/St.Louis/630/09 -----CCNR**S**IISIWASHSIQTGSQNNTGICNQRIITYENSTWVNHTYVNINNTNVVAGE 77

A/St.Louis/792/09 -----CCNR**S**IISIWASHSIQTGSQNNTGICNQRIITYENSTWVNHTYVNINNTNVVAGE 77

A/St.Louis/775/09 -----CCNR**S**IISIWASHSIQTGSQNNTGICNQRIITYENSTWVNHTYVNINNTNVVAGE 77

A/St.Louis/690/09 -----CCNR**S**IISIWASHSIQTGSQNNTGICNQRIITYENSTWVNHTYVNINNTNVVAGE 77

A/St.Louis/539/09 -----CCNR**S**IISIWASHSIQTGSQNNTGICNQRIITYENSTWVNHTYVNINNTNVVAGE 77

A/St.Louis/758/09 -----CCNRNIISIWASHSIQTGSQNNTGICNQRIITYENSTWVNHTYVNINNTNVVAGE 77

A.St.Louis/746/09 -----CCNR**S**IISIWASHSIQTGSQNNTGICNQRIITYENSTWVNHTYVNINNTNVVAGE 77

****.**************************************************

A/St.Louis/790/09 DKTSVTLAGNSSLCSISGWAIYTKDNSIRIGSKGDVFVIREPFISCSHLECRTFFLTQGA 138

A/St.Louis/764/09 DKTSVTLAGNSSLCSISGWAIYTKDNSIRIGSKGDVFVIREPFISCSHLECRTFFLTQGA 138

A/St.Louis/630/09 DKTSVTLAGNSSLCSISGWAIYTKDNSIRIGSKGDVFVIREPFISCSHLECRTFFLTQGA 138

A/St.Louis/792/09 DKTSVTLAGNSSLCSISGWAIYTKDNSIRIGSKGDVFVIREPFISCSHLECRTFFLTQGA 138

A/St.Louis/775/09 DKTSVTLAGNSSLCSISGWAIYTKDNSIRIGSKGDVFVIREPFISCSHLECRTFFLTQGA 138

A/St.Louis/690/09 DKTSVTLAGNSSLCSISGWAIYTKDNSIRIGSKGDVFVIREPFISCSHLECRTFFLTQGA 138

A/St.Louis/539/09 DKTSVTLAGNSSLCSISGWAIYTKDNSIRIGSKGDVFVIREPFISCSHLECRTFFLTQGA 138

A/St.Louis/758/09 DKTSVTLAGNSSLCSISGWAIYTKDNSIRIGSKGDVFVIREPFISCSHLECRTFFLTQGA 138

A.St.Louis/746/09 DKTSVTLAGNSSLCSISGWAIYTKDNSIRIGSKGDVFVIREPFISCSHLECRTFFLTQGA 138

************************************************************

A/St.Louis/790/09 LLNDKHSNGTVKDRSPYRALMSCPLGEAPSPYNSKFESVAWSASACHDGMGWLTIGISGP 197

A/St.Louis/764/09 LLNDKHSNGTVKDRSPYRALMSCPLGEAPSPYNSKFESVAWSASACHDGMGWLTIGISGP 197

A/St.Louis/630/09 LLNDKHSNGTVKDRSPYRALMSCPLGEAPSPYNSKFESVAWSASACHDGMGWLTIGISGP 197

A/St.Louis/792/09 LLNDKHSNGTVKDRSPYRALMSCPLGEAPSPYNSKFESVAWSASACHDGMGWLTIGISGP 197

A/St.Louis/775/09 LLNDKHSNGTVKDRSPYRALMSCPLGEAPSPYNSKFESVAWSASACHDGMGWLTIGISGP 197

A/St.Louis/690/09 LLNDKHSNGTVKDRSPYRALMSCPLGEAPSPYNSKFESVAWSASACHDGMGWLTIGISGP 197

A/St.Louis/539/09 LLNDKHSNGTVKDRSPYRALMSCPLGEAPSPYNSKFESVAWSASACHDGMGWLTIGISGP 197

A/St.Louis/758/09 LLNDKHSNGTVKDRSPYRALMSCPLGEAPSPYNSKFESVAWSASACHDGMGWLTIGISGP 197

A.St.Louis/746/09 LLNDKHSNGTVKDRSPYRALMSCPLGEAPSPYNSKFESVAWSASACHDGMGWLTIGISGP 197

************************************************************

A/St.Louis/790/09 DNGAVAVLKYNGIITGTIKSWKKQILRTQESECVCMNGSCFTIMTDGPSNKAASYKIFKI 257

A/St.Louis/764/09 DNGAVAVLKYNGIITGTIKSWKKQILRTQESECVCMNGSCFTIMTDGPSNKAASYKIFKI 257

A/St.Louis/630/09 DNGAVAVLKYNGIITGTIKSWKKQ**V**LRTQESECVCMNGSCFTIMTDGPSNKAASYKIFKI 257

A/St.Louis/792/09 DNGAVAVLKYNGIITGTIKSWKKQILRTQESECVCMNGSCFTIMTDGPSNKAASYKIFKI 257

A/St.Louis/775/09 DNGAVAVLKYNGIITGTIKSWKKQILRTQESECVCMNGSCFTIMTDGPSNKAASYKIFKI 257

A/St.Louis/690/09 DNGAVAVLKYNGIITGTIKSWKKQ**V**LRTQESECVCMNGSCFTIMTDGPSNKAASYKIFKI 257

A/St.Louis/539/09 DNGAVAVLKYNGIITGTIKSWKKQILRTQESECVCMNGSCFTIMTDGPSNKAASYKIFKI 257

A/St.Louis/758/09 DNGAVAVLKYNGIITGTIKSWKKQILRTQESECVCMNGSCFTIMTDGPSNKAASYKIFKI 257

A.St.Louis/746/09 DNGAVAVLKYNGIITGTIKSWKKQILRTQESECVCMNGSCFTIMTDGPSNKAASYKIFKI 257

************************:***********************************

A/St.Louis/790/09 EKGKVTKSIELNAPNF**Y**YEECSCYPDTGIVMCVCRDNWHGSNRPWVSFNQNLDYQIGYIC 318

A/St.Louis/764/09 EKGKVTKSIELNAPNF**Y**YEECSCYPDTGIVMCVCRDNWHGSNRPWVSFNQNLDYQIGYIC 318

A/St.Louis/630/09 EKGKVTKSIELNAPNF**Y**YEECSCYPDTGIVMCVCRDNWHGSNRPWVSFNQNLDYQIGYIC 318

A/St.Louis/792/09 EKGKVTKSIELNAPNF**Y**YEECSCYPDTGIVMCVCRDNWHGSNRPWVSFNQNLDYQIGYIC 318

A/St.Louis/775/09 EKGKVTKSIELNAPNF**Y**YEECSCYPDTGIVMCVCRDNWHGSNRPWVSFNQNLDYQIGYIC 318

A/St.Louis/690/09 EKGKVTKSIELNAPNF**Y**YEECSCYPDTGIVMCVCRDNWHGSNRPWVSFNQNLDYQIGYIC 318

A/St.Louis/539/09 EKGKVTKSIELNAPNF**Y**YEECSCYPDTGIVMCVCRDNWHGSNRPWVSFNQNLDYQIGYIC 318

A/St.Louis/758/09 EKGKVTKSIELNAPNF**Y**YEECSCYPDTGIVMCVCRDNWHGSNRPWVSFNQNLDYQIGYIC 318

A.St.Louis/746/09 EKGKVTKSIELNAPNF**Y**YEECSCYPDTGIVMCVCRDNWHGSNRPWVSFNQNLDYQIGYIC 318

************************************************************

A/St.Louis/790/09 SGVFGDNPRPEDGEGSCNPVTVDGANGVKGFSYKYGNGVWIGRTKSNRLRKGFEMIWDPN 381

A/St.Louis/764/09 SGVFGDNPRPEDGEGSCNPVTVDGANGVKGFSYKYGNGVWIGRTKSNRLRKGFEMIWDPN 381

A/St.Louis/630/09 SGVFGDNPRPEDGEGSCNPVTVDGANGVKGFSYKYGNGVWIGRTKSNRLRKGFEMIWDPN 381

A/St.Louis/792/09 SGVFGDNPRPEDGEGSCNPVTVDGANGVKGFSYKYGNGVWIGRTKSNRLRKGFEMIWDPN 381

A/St.Louis/775/09 SGVFGDNPRPEDGEGSCNPVTVDGANGVKGFSYKYGNGVWIGRTKSNRLRKGFEMIWDPN 381

A/St.Louis/690/09 SGVFGDNPRPEDGEGSCNPVTVDGANGVKGFSYKYGNGVWIGRTKSNRLRKGFEMIWDPN 381

A/St.Louis/539/09 SGVFGDNPRPEDGEGSCNPVTVDGANGVKGFSYKYGNGVWIGRTKSNRLRKGFEMIWDPN 381

A/St.Louis/758/09 SGVFGDNPRPEDGEGSCNPVTVDGANGVKGFSYKYGNGVWIGRTKSNRLRKGFEMIWDPN 381

A.St.Louis/746/09 SGVFGDNPRPEDGEGSCNPVTVDGANGVKGFSYKYGNGVWIGRTKSNRLRKGFEMIWDPN 381

************************************************************

A/St.Louis/790/09 GWTNTDSDFSVKQDVVAITDWSGYSGSFVQHPELTGLDCIRPCFWVELVRGLPRENTTIW 438

A/St.Louis/764/09 GWTNTDSDFSVKQDVVAITDWSGYSGSFVQHPELTGLDCIRPCFWVELVRGLPRENTTIW 438

A/St.Louis/630/09 GWTNTDSDFSVKQDVVAITDWSGYSGSFVQHPELTGLDCIRPCFWVELVRGLPRENTTIW 438

A/St.Louis/792/09 GWTNTDSDFSVKQDVVAITDWSGYSGSFVQHPELTGLDCIRPCFWVELVRGLPRENTTIW 438

A/St.Louis/775/09 GWTNTDSDFSVKQDVVAITDWSGYSGSFVQHPELTGLDCIRPCFWVELVRGLPRENTTIW 438

A/St.Louis/690/09 GWTNTDSDFSVKQDVVAITDWSGYSGSFVQHPELTGLDCIRPCFWVELVRGLPRENTTIW 438

A/St.Louis/539/09 GWTNTDSDFSVKQDVVAITDWSGYSGSFVQHPELTGLDCIRPCFWVELVRGLPRENTTIW 438

A/St.Louis/758/09 GWTNTDSDFSVKQDVVAITDWSGYSGSFVQHPELTGLDCIRPCFWVELVRGLPRENTTIW 438

A.St.Louis/746/09 GWTNTDSDFSVKQDVVAITDWSGYSGSFVQHPELTGLDCIRPCFWVELVRGLPRENTTIW 438

************************************************************

A/St.Louis/790/09 TSGSSISFCGVNSDTA 454

A/St.Louis/764/09 TSGSSISFCGVNSDTA 454

A/St.Louis/630/09 TSGSSISFCGVNSDTA 454

A/St.Louis/792/09 TSGSSISFCGVNSDTA 454

A/St.Louis/775/09 TSGSSISFCGVNSDTA 454

A/St.Louis/690/09 TSGSSISFCGVNSDTA 454

A/St.Louis/539/09 TSGSSISFCGVNSDTA 454

A/St.Louis/758/09 TSGSSISFCGVNSDTA 454

A.St.Louis/746/09 TSGSSISFCGVNSDTA 454

****************
