# Supplementary material for: Inhibition of Neuraminidase Inhibitor-Resistant Influenza Virus by DAS181, a Novel Sialidase Fusion Protein
Source: PLoS One. 2009 Nov 6;4(11):e7838. doi: 10.1371/journal.pone.0007838 (PMC2770896; doi:10.1371/journal.pone.0007838)
Supplement: Figure S4 — NA alignment of recently published H1N1 IFV isolates. Published HA sequences for several 2007/2008 isolates were aligned with Clustal W2 software to determine the conservation of amino acid choice at select regions identified as mutations in Figure S1. Sequence data noted with N2 numbering scheme, as previously described [41]. Highlighted residues correspond to: Red = N32, Green = I222, Blue = H274. * = identical amino acid, : = highly similar amino acid, . = moderately similar amino acid. Accession numbers for NA sequences aligned here: A/Kentucky/UR06-0369/2007 = CY037665 A/Texas/UR06-0422/2007 = CY037441 A/Ohio/UR06-0493/2007 = CY037657 A/NewJersey/15/2007 = EU516148 A/Perth/33/2008 = FJ743472 A/Kentucky/UR07-0061/2008 = CY037697 A/Washington/AF06/2007 = CY037329 A/Florida/UR07-0022/2008 = CY037681 A/Hawaii/21/2007 = EU516112 A/Japan/AF07/2008 = CY037337 A/Cambodia/21/2007 = FJ743470 A/Tennessee/UR06-0106/2007 = CY037785 A/Vermont/UR06-0513/2007 = CY037465 (0.05 MB DOC) [file pone.0007838.s005.doc]

A/NewJersey/15/2007 ------MNPNQKIITIGSISIAIGIISLMLQIGNIISIWASHSIQTGSQN 48

A/Perth/33/2008 QKQEFKMNPNQKIITIGSISIAIGIISLMLQIGNIISIWASHSIQTGSQN 48

A/Hawaii/21/2007 ------MNPNQKIITIGSISIAIGIISLMLQIGNIISIWASHSIQTGSQN 48

A/Japan/AF07/2008 -------NPNQKIITIGSISIAIGIISLMLQIGNIISIWASHSIQTGSQN 48

A/Washington/AF06/2007 ---------NQKIITIGSISIAIGIISLMLQIGNIISIWASHSIQTGSQN 48

A/Kentucky/UR07-0061/2008 ------MNPNQKIITIGSISIAIGIISLMLQIGNIISIWASHSIQTGSQN 48

A/Florida/UR07-0022/2008 ------MNPNQKIITIGSISIAIGIISLMLQIGNIISIWASHSIQTGSQN 48

A/Cambodia/21/2007 QKQEFKMNPNQKIITIGSISIAIGIISLMLQIGNIISIWASHSIQTGSQN 48

A/Kentucky/UR06-0369/2007 ------MNPNQKIITIGSISIAIGIISLMLQIGNIISIWASHSIQTGSQN 48

A/Texas/UR06-0422/2007 ---------NQKIITIGSISIAIGIISLMLQIGNIISIWASHSIQTGSQN 48

A/Vermont/UR06-0513/2007 ------MNPNQKIITIGSISIAIGIISLMLQIGNIISIWASHSIQTGSQN 48

A/Ohio/UR06-0493/2007 ------MNPNQKIITIGSISIAIGIISLMLQIGNIISIWASHSIQTGSQN 48

A/Tennessee/UR06-0106/2007 ------MNPNQKIITIGSISIAIGIISLMLQIGNIISIWASHSIQTGSQN 48

*****************************************

A/NewJersey/15/2007 NTGICNQRIITYENSTWVNHTYVNINNTNVVAGEDKTSVTLAGNSSLCSI 94

A/Perth/33/2008 NTGICNQRIITYENSTWVNHTYVNINNTNVVAGEDKTSVTLAGNSSLCSI 94

A/Hawaii/21/2007 NTGICNQRIITYENSTWVNHTYVNINNTNVVAGEDKTSVTLAGNSSLCSI 94

A/Japan/AF07/2008 NTGICNQRIITYENSTWVNHTYVNINNTNVVAGEDKTSVTLAGNSSLCSI 94

A/Washington/AF06/2007 NTGICNQRIITYENSTWVNHTYVNINNTNVVAGEDKTSVTLAGNSSLCSI 94

A/Kentucky/UR07-0061/2008 NTGICNQRIITYENSTWVNHTYVNINNTNVVAGEDKTSVTLAGNSSLCSI 94

A/Florida/UR07-0022/2008 NTGICNQRIITYENSTWVNHTYVNINNTNVVAGEDKTSVTLAGSSSLCSI 94

A/Cambodia/21/2007 HTGICNQRIITYENSTWVNHTYVNINNTNVVAGKDKTPVTLAGNSSLCSI 94

A/Kentucky/UR06-0369/2007 HTGICNQRIITYENSTWVNHTYVSINNTNVVAGKDKTSVTLAGNSSLCSI 94

A/Texas/UR06-0422/2007 HTGICNQRIITYENSTWVNHTYVSINNTNVVAGKDKTSVTLAGNSSLCSI 94

A/Vermont/UR06-0513/2007 HTGICNQRIITYENSTWVNHTYVSINNTNVVAGKDKTSVTLAGNSSLCSI 94

A/Ohio/UR06-0493/2007 HTGICNQRIITYENSTWVNHTYVSINNTNVVAGKDKTSVTLAGNSSLCSI 94

A/Tennessee/UR06-0106/2007 HTGICNQRIITYENSTWVNHTYVNINNTNVVAGKDKTSVTLAGNSSLCSI 94

:**********************.*********:***.*****.******

A/NewJersey/15/2007 SGWAIYTKDNSIRIGSKGDVFVIREPFISCSHLECRTFFLTQGALLNDKH 144

A/Perth/33/2008 SGWAIYTKDNSIRIGSKGDVFVIREPFISCSHLECRTFFLTQGALLNDKH 144

A/Hawaii/21/2007 SGWAIYTKDNSIRIGSKGDVFVIREPFISCSHLECRTFFLTQGALLNDKH 144

A/Japan/AF07/2008 SGWAIYTKDNSIRIGSKGDVFVIREPFISCSHLECRTFFLTQGALLNDKH 144

A/Washington/AF06/2007 SGWAIYTKDNSIRIGSKGDVFVIREPFISCSHLECRTFFLTQGALLNDKH 144

A/Kentucky/UR07-0061/2008 SGWAIYTKDNSIRIGSKGDVFVIREPFISCSHLECRTFFLTQGALLNDKH 144

A/Florida/UR07-0022/2008 SGWAIYTKDNSIRIGSKGDVFVIREPFISCSHLECRTFFLTQGALLNDKH 144

A/Cambodia/21/2007 SGWAIHTKDNSIRIGSKGDVFVIREPFISCSHLECKTFFLTQGALLNDKH 144

A/Kentucky/UR06-0369/2007 SGWAIYTKDNSIRIGSKGDVFVIREPFISCSHLECRTFFLTQGALLNDKH 144

A/Texas/UR06-0422/2007 SGWAIYTKDNSIRIGSKGDVFVIREPFISCSHLECRTFFLTQGALLNDKH 144

A/Vermont/UR06-0513/2007 SGWAIYTKDNSIRIGSKGDVFVIREPFISCSHLECRTFFLTQGALLNDKH 144

A/Ohio/UR06-0493/2007 SGWAIYTKDNSIRIGSKGDVFVIREPFISCSHLECRTFFLTQGALLNDKH 144

A/Tennessee/UR06-0106/2007 SGWAIYTKDNSIRIGSKGDVFVIREPFISCSHLECRTFFLTQGALLNDKH 144

*****:*****************************:**************

A/NewJersey/15/2007 SNGTVKDRSPYRALMSCPLGEAPSPYNSKFESVAWSASACHDGMGWLTIG 193

A/Perth/33/2008 SNGTVKDRSPYRALMSCPLGEAPSPYNSKFESVAWSASACHDGMGWLTIG 193

A/Hawaii/21/2007 SNGTVKDRSPYRALMSCPLGEAPSPYNSKFESVAWSASACHDGMGWLTIG 193

A/Japan/AF07/2008 SNGTVKDRSPYRALMSCPLGEAPSPYNSKFESVAWSASACHDGMGWLTIG 193

A/Washington/AF06/2007 SNGTVKDRSPYRALMSCPLGEAPSPYNSKFESVAWSASACHDGMGWLTIG 193

A/Kentucky/UR07-0061/2008 SNGTVKDRSPYRALMSCPLGEAPSPYNSKFESVAWSASACHDGMGWLTIG 193

A/Florida/UR07-0022/2008 SNGTVKDRSPYRALMSCPLGEAPSPYNSKFESVAWSASACHDGMGWLTIG 193

A/Cambodia/21/2007 SNGTVKDRSPYRALMSCPLGEAPSPYNSKFESVAWSASACHDGIGWLTIG 193

A/Kentucky/UR06-0369/2007 SNGTVKDRSPYRALMSCPLGEAPSPYNSKFESVAWSASACHDGMGWLTIG 193

A/Texas/UR06-0422/2007 SNGTVKDRSPYRALMSCPLGEAPSPYNSKFESVAWSASACHDGMGWLTIG 193

A/Vermont/UR06-0513/2007 SNGTVKDRSPYRALMSCPLGEAPSPYNSKFESVAWSASACHDGMGWLTIG 193

A/Ohio/UR06-0493/2007 SNGTVKDRSPYRALMSCPLGEAPSPYNSKFESVAWSASACHDGMGWLTIG 193

A/Tennessee/UR06-0106/2007 SNGTVKDRSPYRALMSCPLGEAPSPYNSKFESVAWSASACHDGMGWLTIG 193

*******************************************:******

A/NewJersey/15/2007 ISGPDNGAVAVLKYNGIITGTIKSWKKQILRTQESECVCMNGSCFTIMTD 243

A/Perth/33/2008 ISGPDNGAVAVLKYNGIITGTIKSWKKQILRTQESECVCMNGSCFTIMTD 243

A/Hawaii/21/2007 ISGPDNGAVAVLKYNGIITGTIKSWKKQILRTQESECVCMNGSCFTIMTD 243

A/Japan/AF07/2008 ISGPDNGAVAVLKYNGIITGTIKSWKKQILRTQESECVCMNGSCFTIMTD 243

A/Washington/AF06/2007 ISGPDNGAVAVLKYNGIITGTIKSWKKQILRTQESECVCMNGSCFTIMTD 243

A/Kentucky/UR07-0061/2008 ISGPDNGAVAVLKYNGIITGTIKSWKKQILRTQESECVCMNGSCFTIMTD 243

A/Florida/UR07-0022/2008 ISGPDNGAVAVLKYNGIITGTIKSWKKQILRTQESECVCMNGSCFTIMTD 243

A/Cambodia/21/2007 ISGPDNGAVAVLKYNGIITGTIKSWKKQILRTQESECVCMNGSCFTIMTD 243

A/Kentucky/UR06-0369/2007 ISGPDNGAVAVLKYNGIITETIKSWKKRILRTQESECVCVNGSCFTIMTD 243

A/Texas/UR06-0422/2007 ISGPDNGAVAVLKYNGIITETIKSWKKRILRTQESECVCVNGSCFTIMTD 243

A/Vermont/UR06-0513/2007 ISGPDNGAVAVLKYNGIITETIKSWKKRILRTQESECVCVNGSCFTIMTD 243

A/Ohio/UR06-0493/2007 ISGPDNGAVAVLKYNGIITGTIKSWKKRILRTQESECVCVNGSCFTIMTD 243

A/Tennessee/UR06-0106/2007 ISGPDNGAVAVLKYNGIITETIKSWKKRILRTQESECVCVNGSCFTIMTD 243

******************* *******:***********:**********

A/NewJersey/15/2007 GPSNKAASYKIFKIEKGKVTKSIELNAPNF**Y**YEECSCYPDTGIVMCVCRD 293

A/Perth/33/2008 GPSNKAASYKIFKIEKGKVTKSIELNAPNF**Y**YEECSCYPDTGIVMCVCRD 293

A/Hawaii/21/2007 GPSNKAASYKIFKIEKGKVTKSIELNAPNF**Y**YEECSCYPDTGIVMCVCRD 293

A/Japan/AF07/2008 GPSNKAASYKIFKIEKGKVTKSIELNAPNFHYEECSCYPDTGIVMCVCRD 293

A/Washington/AF06/2007 GPSNKAASYKIFKIEKGKVTKSIELNAPNFHYEECSCYPDTGIVMCVCRD 293

A/Kentucky/UR07-0061/2008 GPSNKAASYKIFKIEKGKVTKSIELNAPNFHYEECSCYPDTGIVMCVCRD 293

A/Florida/UR07-0022/2008 GPSNKAASYKIFKIEKGKVTKSIELNAPNFHYEECSCYPDTGIVMCVCRD 293

A/Cambodia/21/2007 GPSNGAASYKIFKIEKGKVTKSMELNAPNF**Y**YEECSCYPDTGTVMCVCRD 293

A/Kentucky/UR06-0369/2007 GPSNGAASYKIFKIEKGKVTKSIELNAPNFHYEECSCYPDTGTVMCVCRD 293

A/Texas/UR06-0422/2007 GPSNGAASYKIFKIEKGKVTKSIELNAPNFHYEECSCYPDTGTVMCVCRD 293

A/Vermont/UR06-0513/2007 GPSNGAASYKIFKIEKGKVTKSIELNAPNFHYEECSCYPDTGTVMCVCRD 293

A/Ohio/UR06-0493/2007 GPSNGAASYKIFKIEKGKVTKSIELNAPNFHYEECSCYPDTGTVMCVCRD 293

A/Tennessee/UR06-0106/2007 GPSNGAASYKIFKIEKGKVTKSIELNAPNFHYEECSCYPDTGTVMCVCRD 293

**** *****************:*******:*********** *******

A/NewJersey/15/2007 NWHGSNRPWVSFNQNLDYQIGYICSGVFGDNPRPEDGEGSCNPVTVDGAN 345

A/Perth/33/2008 NWHGSNRPWVSFNQNLDYQIGYICSGVFGDNPRPEDGEGSCNPVTVDGAN 345

A/Hawaii/21/2007 NWHGSNRPWVSFNQNLDYQIGYICSGVFGDNPRPEDGEGSCNPVTVDGAN 345

A/Japan/AF07/2008 NWHGSNRPWVSFNQNLDYQIGYICSGVFGDNPRPEDGEGSCNPVTVDGAN 345

A/Washington/AF06/2007 NWHGSNRPWVSFNQNLDYQIGYICSGVFGDNPRPEDGEGSCNPVTVDGAN 345

A/Kentucky/UR07-0061/2008 NWHGSNRPWVSFNQNLDYQIGYICSGVFGDNPRPEDGEGSCNPVTVDGAN 345

A/Florida/UR07-0022/2008 NWHGSNRPWVSFNQNLDYQIGYICSGVFGDNPRPEDGEGSCNPVTVDGAN 345

A/Cambodia/21/2007 NWHGSNRPWVSFNQNLDYQIGYICSGVFGDNPRPEDGEGSCNPVTVDGAN 345

A/Kentucky/UR06-0369/2007 NWHGSNRPWVSFNQNLDYQIGYICSGVFGDNPRPKDGKGSCNPVTVDGAD 345

A/Texas/UR06-0422/2007 NWHGSNRPWVSFNQNLDYQIGYICSGVFGDNPRPKDGKGSCNPVTVDGAD 345

A/Vermont/UR06-0513/2007 NWHGSNRPWVSFNQNLDYQIGYICSGVFGDNPRPKDGKGSCNPVTVDGAD 345

A/Ohio/UR06-0493/2007 NWHGSNRPWVSFNQNLDYQIGYICSGVFGDNPRPKDGKGSCNPVTVDGAD 345

A/Tennessee/UR06-0106/2007 NWHGSNRPWVSFNQNLDYQIGYICSGVFGDNPRPKDGKGSCNPVTVDGAD 345

**********************************:**:***********:

A/NewJersey/15/2007 GVKGFSYKYGNGVWIGRTKSNRLRKGFEMIWDPNGWTNTDSDFSVKQDVV 398

A/Perth/33/2008 GVKGFSYKYGNGVWIGRTKSNRLRKGFEMIWDPNGWTNTDSDFSVKQDVV 398

A/Hawaii/21/2007 GVKGFSYKYDNGVWIGRTKSNRLRKGFEMIWDPNGWTNTDSDFSVKQDVV 398

A/Japan/AF07/2008 GVKGFSYKYDNGVWIGRTKSNRLRKGFEMIWDPNGWTNTDSDFSVKQDVV 398

A/Washington/AF06/2007 GVKGFSYKYDNGVWIGRTKSNRLRKGFEMIWDPNGWTNTDSDFSVKQDVV 398

A/Kentucky/UR07-0061/2008 GVKGFSYKYDNGVWIGRTKSNRLRKGFEMIWDPNGWTNTDSDFSVKQDVV 398

A/Florida/UR07-0022/2008 GVKGFSYKYDNGVWIGRTKSNRLRKGFEMIWDPNGWTNTDSDFSVKQDVV 398

A/Cambodia/21/2007 GVKGFSYKYGNGVWIGRTKSNRIRKGFEMIWDPNGWTNTDSDFSVKQDIV 398

A/Kentucky/UR06-0369/2007 GVKGFSYKYGNGVWIGRTKSNRLRKGFEMIWDPNGWTDTDSDFSVKQDVV 398

A/Texas/UR06-0422/2007 GVKGFSYKYGNGVWIGRTKSNRLRKGFEMIWDPNGWTDTDSDFSVKQDVV 398

A/Vermont/UR06-0513/2007 GVKGFSYKYGNGVWIGRTKSNRLRKGFEMIWDPNGWTDTDSDFSVKQDVV 398

A/Ohio/UR06-0493/2007 GVKGFSYKYGNGVWIGRTKSNRLRKGFEMIWDPNGWTDTDSDFSVKQDVV 398

A/Tennessee/UR06-0106/2007 GVKGFSYKYGNGVWIGRTKSNRLRKGFEMIWDPNGWTNTDSDFSVKQDVV 398

*********.************:**************:**********:*

A/NewJersey/15/2007 AITDWSGYSGSFVQHPELTGLDCIRPCFWVELVRGLPRENTTIWTSGSSI 444

A/Perth/33/2008 AITDWSGYSGSFVQHPELTGLDCIRPCFWVELVRGLPRENTTIWTSGSSI 444

A/Hawaii/21/2007 AITDWSGYSGSFVQHPELTGLDCIRPCFWVELVRGLPRENTTIWTSGSSI 444

A/Japan/AF07/2008 AITDWSGYSGSFVQHPELTGLDCIRPCFWVELVRGLPRENTTIWTSGSSI 444

A/Washington/AF06/2007 AITDWSGYSGSFVQHPELTGLDCIRPCFWVELVRGLPRENTTIWTSGSSI 444

A/Kentucky/UR07-0061/2008 AITDWSGYSGSFVQHPELTGLDCIRPCFWVELVRGLPRENTTIWTSGSSI 444

A/Florida/UR07-0022/2008 AITDWSGYSGSFVQHPELTGLDCIRPCFWVELVRGLPRENTTIWTSGSSI 444

A/Cambodia/21/2007 AITDWSGYSGSFVQHPELTGLDCIRPCFWVELVRGLPRENTTIWTSGSSI 444

A/Kentucky/UR06-0369/2007 AITDWSGYSGSFVQHPELTGLDCIRPCFWVELVRGLPRENTTIWTSGSSI 444

A/Texas/UR06-0422/2007 AITDWSGYSGSFVQHPELTGLDCIRPCFWVELVRGLPRENTTIWTSGSSI 444

A/Vermont/UR06-0513/2007 AITDWSGYSGSFVQHPELTGLDCIRPCFWVELVRGLPRENTTIWTSGSSI 444

A/Ohio/UR06-0493/2007 AITDWSGYSGSFVQHPELTGLDCIRPCFWVELVRGLPRENTTIWTSGSSI 444

A/Tennessee/UR06-0106/2007 AITDWSGYSGSFVQHPELTGLDCIRPCFWVELVRGLPRENTTIWTSGSSI 444

**************************************************

A/NewJersey/15/2007 SFCGVNSDTANWSWPDGAELPFTIDK 469

A/Perth/33/2008 SFCGVNSDTANWSWPDGAELPFTIDK 469

A/Hawaii/21/2007 SFCGVNSDTANWSWPDGAELPFTIDK 469

A/Japan/AF07/2008 SFCGVNSDTANWSWPDGAELPFTIDK 469

A/Washington/AF06/2007 SFCGVNSDTANWSWPDGAELPFTIDK 469

A/Kentucky/UR07-0061/2008 SFCGVNSDTANWSWPDGAELPFTIDK 469

A/Florida/UR07-0022/2008 SFCGVNSDTANWSWPDGAELPFTIDK 469

A/Cambodia/21/2007 SFCGVNSDIANWSWPDGAELPFTIDK 469

A/Kentucky/UR06-0369/2007 SFCGVDSDTANWSWPDGAELPFTIDK 469

A/Texas/UR06-0422/2007 SFCGVDSDTANWSWPDGAELPFTIDK 469

A/Vermont/UR06-0513/2007 SFCGVDSDTANWSWPDGAELPFTIDK 469

A/Ohio/UR06-0493/2007 SFCGVDSDTANWSWPDGAELPFTIDK 469

A/Tennessee/UR06-0106/2007 SFCGVDSDTANWSWPDGAELPFTIDK 469

*****:** *****************
