# Supplementary material for: Inhibition of Neuraminidase Inhibitor-Resistant Influenza Virus by DAS181, a Novel Sialidase Fusion Protein
Source: PLoS One. 2009 Nov 6;4(11):e7838. doi: 10.1371/journal.pone.0007838 (PMC2770896; doi:10.1371/journal.pone.0007838)
Supplement: Figure S5 — HA alignment of all 2009 isolates tested here. Primers designed to clone the entire HA gene were used to also sequence the HA gene. Data was obtained for the entire HA gene except the region corresponding to the first 25–31 and final 12 amino acids. Sequences were aligned with Clustal W2 software. Sequence data noted with H3 numbering scheme, as previously described [42]. Highlighted residues correspond to: Red = N163, Green = G/V189, Blue = D225. * = identical amino acid, : = highly similar amino acid, . = moderately similar amino acid. Poor data was obtained for isolate 690 in region of first 100 amino acids so this sequence is omitted from alignment comparison here. Accession numbers for HA sequences aligned here: A/St.Louis/790/2009 = GQ994961 A/St.Louis/764/2009 = GQ994959 A/St.Louis/630/2009 = GQ994955 A/St.Louis/792/2009 = GQ994962 A/St.Louis/775/2009 = GQ994960 A/St.Louis/690/2009 = GQ994956 A/St.Louis/539/2009 = GQ994954 A/St.Louis/758/2009 = GQ994958 A/St.Louis/746/2009 = GQ994957 (0.04 MB DOC) [file pone.0007838.s006.doc]

A/St.Louis/539/09 ---------VDTVLEKNVTVTHSVNLLENSHNGKLCLLKGIAPLQLGNCSVAGWILGNPE 75

A/St.Louis/758/09 ---------VDTVLEKNVTVTHSVNLLENSHNGKLCLLKGIAPLQLGNCSVAGWILGNPE 75

A/St.Louis/630/09 ---------VDTVLEKNVTVTHSVNLLENSHNGKLCLLKGIAPLQLGNCSVAGWILGNPE 75

A/St.Louis/792/09 ---------VDTVLEKNVTVTHSVNLLENSHNGKLCLLKGIAPLQLGNCSVAGWILGNPE 75

A/St.Louis/775/09 ---------VDTVLEKNVTVTHSVNLLENSHNGKLCLLKGIAPLQLGNCSVAGWILGNPE 75

A/St.Louis/790/09 ---------VDTVLEKNVTVTHSVNLLENSHNGKLCLLKGIAPLQLGNCSVAGWILGNPE 75

A/St.Louis/764/09 ---------VDTVLEKNVTVTHSVNLLENSHNGKLCLLKGIAPLQLGNCSVAGWILGNPE 75

A/St.Louis/746/09 ---------------KNVTVTHSVNLLEKAR-GKLCLLKGIAPLQLGNCSVAGWILGNPE 75

A/St.Louis/690/09 ------------------------------------------------------------ 75

*************::: ****************************

A/St.Louis/539/09 CELLISKESWSYIVEKPNPENGTCYPGHFADYEELREQLSSVSSFERFEIFPKESSWPNH 130

A/St.Louis/758/09 CELLISKESWSYIVEKPNPENGTCYPGHFADYEELREQLSSVSSFERFEIFPKESSWPNH 130

A/St.Louis/630/09 CELLISKESWSYIVEKPNPENGTCYPGHFADYEELREQLSSVSSFERFEIFPKESSWPNH 130

A/St.Louis/792/09 CELLISKESWSYIVEKPNPENGTCYPGHFADYEELREQLSSVSSFERFEIFPKESSWPNH 130

A/St.Louis/775/09 CELLISKESWSYIVEKPNPENGTCYPGHFADYEELREQLSSVSSFERFEIFPKESSWPNH 130

A/St.Louis/790/09 CELLISKESWSYIVEKPNPENGTCYPGHFADYEELREQLSSVSSFERFEIFPKESSWPNH 130

A/St.Louis/764/09 CELLISKESWSYIVEKPNPENGTCYPGHFADYEELREQLSSVSSFERFEIFPKESSWPNH 130

A/St.Louis/746/09 CELLISKESWSYIVEKPNPENGTCYPGHFADYEELREQLSSVSSFERFEIFPKESSWPNH 130

A/St.Louis/690/09 ------------------------------------------------------------ 130

************************************************************

A/St.Louis/539/09 TVTGVSASCSHNGESSFYRNLLWLTGKNGLYPNLSKSYANNKEKEVLVLWGVHHPPNIVD 190

A/St.Louis/758/09 TVTGVSASCSHNGENSFYRNLLWLTGKNGLYP**T**LSKSYANNKEKEVLVLWGVHHPPNI**A**D 190

A/St.Louis/630/09 TVTGVSASCSHNGESSFYRNLLWLTGKNGLYPNLSKSYANNKEKEVLVLWGVHHPPNIVN 190

A/St.Louis/792/09 TVTGVSASCSHNGESSFYRNLLWLTGKNGLYPNLSKSYANNKEKEVLVLWGVHHPPNIVD 190

A/St.Louis/775/09 TVTGVSASCSHNGESSFYRNLLWLTGKNGLYPNLSKSYANNKEKEVLVLWGVHHPPNIVN 190

A/St.Louis/790/09 TVTGVSASCSHNGESSFYRNLLWLTGKNGLYPNLSKSYANNKEKEVLVLWGVHHPPNIVN 190

A/St.Louis/764/09 TVTGVSASCSHNGESSFYRNLLWLTGKNGLYP**H**LSKSYANNKEKEVLVLWGVHHPPNIVD 190

A/St.Louis/746/09 TVTGVSASCSHNGESSFYRNLLWLTGKNGLYPNLSKSYANNKEKEVLVLWGVHHPPNIVN 190

A/St.Louis/690/09 TVTGVSASCSHNGESSFYRNLLWLTGKNGLYPNLSKSYANNKEKEVLVLWGVHHPPNIVV 190

**************.***************** *************************.

A/St.Louis/539/09 QKTLYRTENAYVSVVSSHYSRKFTPEIAKRPKVR**G**QEGRINYYWTLLEPGDTIIFEANGN 250

A/St.Louis/758/09 QKTLYHTENAYVSVVSSHYSRKFTPEIAKRPKVRDQEGRINYYWTLLEPGDTIIFEANGN 250

A/St.Louis/630/09 QKTLYRTENAYVSVVSSHYSRKFTPEIAKRPKVRDQEGRINYYWTLLEPGDTIIFEANGN 250

A/St.Louis/792/09 QKTLYRTENAYVSVVSSHYSRKFTPEIAKRPKVR**G**QEGRINYYWTLLEPGDTIIFEANGN 250

A/St.Louis/775/09 QKTLYRTENAYVSVVSSHYSRKFTPEIAKRPKVRDQEGRINYYWTLLEPGDTIIFEANGN 250

A/St.Louis/790/09 QKTLYRTENAYVSVVSSHYSRKFTPEIAKRPKVRDQEGRINYYWTLLEPGDTIIFEANGN 250

A/St.Louis/764/09 QKTLYRTENAYVSVVSSHYSRKFTPEIAKRPKVRDQEGRINYYWTLLEPGDTIIFEANGN 250

A/St.Louis/746/09 QKTLYRTENAYVSVVSSHYSRKFTPEIAKRPKVRDQEGRINYYWTLLEPGDTIIFEANGN 250

A/St.Louis/690/09 QKTLYRTENAYVSVVSSHYSRKFTPEIAKRPKVRDQEGRINYYWTLLEPGDTIIFEANGN 250

*****:****************************.*************************

A/St.Louis/539/09 LIAPRYAFALSRGFGSGIINSNAPMGKCDAKCQTPQGAINSSLPFQNVHPVTIGECPKYV 309

A/St.Louis/758/09 LIAPRYAFALSRGFGSGIINSNAPMDKCDAKCQTPQGAINSSLPFQNVHPVTIGECPKYV 309

A/St.Louis/630/09 LIAPRYAFALSRGFGSGIINSNAPMGKCDAKCQTPQGAINSSLPFQNVHPVTIGECPKYV 309

A/St.Louis/792/09 LIAPRYAFALSRGFGSGIINSNAPMGKCDAKCQTPQGAINSSLPFQNVHPVTIGECPKYV 309

A/St.Louis/775/09 LIAPRYAFALSRGFGSGIINSNAPMGKCDAKCQTPQGAINSSLPFQNVHPVTIGECPKYV 309

A/St.Louis/790/09 LIAPRYAFALSRGFGSGIINSNAPMGKCDAKCQTPQGAINSSLPFQNVHPVTIGECPKYV 309

A/St.Louis/764/09 LIAPRYAFALSRGFGSGIINSNAPMGKCDAKCQTPQGAINSSLPFQNVHPVTIGECPKYV 309

A/St.Louis/746/09 LIAPRYAFALSRGFGSGIINSNAPMGKCDAKCQTPQGAINSSLPFQNVHPVTIGECPKYV 309

A/St.Louis/690/09 LIAPRYAFALSRGFGSGIINSNAPMGKCDAKCQTPQGAINSSLPFQNVHPVTIGECPKYV 309

*************************.**********************************

A/St.Louis/539/09 RSAKLRMVTGLRNIPSIQSRGLFGAIAGFIEGGWTGMVDGWYGYHHQNEQGSGYAADQKS 369

A/St.Louis/758/09 RSAKLRMVTGLRNIPSIQSRGLFGAIAGFIEGGWTGMVDGWYGYHHQNEQGSGYAADQKS 369

A/St.Louis/630/09 RSAKLRMVTGLRNIPSIQSRGLFGAIAGFIEGGWTGMVDGWYGYHHQNEQGSGYAADQKS 369

A/St.Louis/792/09 RSAKLRMVTGLRNIPSIQSRGLFGAIAGFIEGGWTGMVDGWYGYHHQNEQGSGYAADQKS 369

A/St.Louis/775/09 RSAKLRMVTGLRNIPSIQSRGLFGAIAGFIEGGWTGMVDGWYGYHHQNEQGSGYAADQKS 369

A/St.Louis/790/09 RSAKLRMVTGLRNIPSIQSRGLFGAIAGFIEGGWTGMVDGWYGYHHQNEQGSGYAADQKS 369

A/St.Louis/764/09 RSAKLRMVTGLRNIPSIQSRGLFGAIAGFIEGGWTGMVDGWYGYHHQNEQGSGYAADQKS 369

A/St.Louis/746/09 RSAKLRMVTGLRNIPSIQSRGLFGAIAGFIEGGWTGMVDGWYGYHHQNEQGSGYAADQKS 369

A/St.Louis/690/09 RSAKLRMVTGLRNIPSIQSRGLFGAIAGFIEGGWTGMVDGWYGYHHQNEQGSGYAADQKS 369

************************************************************

A/St.Louis/539/09 TQNAINGITNKVNSVIEKMNTQFTAVGKEFNKLERRMENLNKKVDDGFIDIWTYNAELLV 429

A/St.Louis/758/09 TQNAINGITNKVNSVIEKMNTQFTAVGKEFNKLERRMENLNKKVDDGFIDIWTYNAELLV 429

A/St.Louis/630/09 TQNAINGITNKVNSVIEKMNTQFTAVGKEFNKLERRMENLNKKVDDGFIDIWTYNAELLV 429

A/St.Louis/792/09 TQNAINGITNKVNSVIEKMNTQFTAVGKEFNKLERRMENLNKKVDDGFIDIWTYNAELLV 429

A/St.Louis/775/09 TQNAINGITNKVNSVIEKMNTQFTAVGKEFNKLERRMENLNKKVDDGFIDIWTYNAELLV 429

A/St.Louis/790/09 TQNAINGITNKVNSVIEKMNTQFTAVGKEFNKLERRMENLNKKVDDGFIDIWTYNAELLV 429

A/St.Louis/764/09 TQNAINGITNKVNSVIEKMNTQFTAVGKEFNKLERRMENLNKKVDDGFIDIWTYNAELLV 429

A/St.Louis/746/09 TQNAINGITNKVNSVIEKMNTQFTAVGKEFNKLERRMENLNKKVDDGFIDIWTYNAELLV 429

A/St.Louis/690/09 TQNAINGITNKVNSVIEKMNTQFTAVGKEFNKLERRMENLNKKVDDGFIDIWTYNAELLV 429

************************************************************

A/St.Louis/539/09 LLENERTLDFHDSNVKNLYEKVKSQLKNNAKEIGNGCFEFYHKCNDECMESVKNGTYDYP 489

A/St.Louis/758/09 LLENERTLDFHDSNVKNLYEKVKSQLKNNAKEIGNGCFEFYHKCNDECMESVKNGTYDYP 489

A/St.Louis/630/09 LLENERTLDFHDSNVKNLYEKVKSQLKNNAKEIGNGCFEFYHKCNDECMESVKNGTYDYP 489

A/St.Louis/792/09 LLENERTLDFHDSNVKNLYEKVKSQLKNNAKEIGNGCFEFYHKCNDECMESVKNGTYDYP 489

A/St.Louis/775/09 LLENERTLDFHDSNVKNLYEKVKSQLKNNAKEIGNGCFEFYHKCNDECMESVKNGTYDYP 489

A/St.Louis/790/09 LLENERTLDFHDSNVKNLYEKVKSQLKNNAKEIGNGCFEFYHKCNDECMESVKNGTYDYP 489

A/St.Louis/764/09 LLENERTLDFHDSNVKNLYEKVKSQLKNNAKEIGNGCFEFYHKCNDECMESVKNGTYDYP 489

A/St.Louis/746/09 LLENERTLDFHDSNVKNLYEKVKSQLKNNAKEIGNGCFEFYHKCNDECMESVKNGTYDYP 489

A/St.Louis/690/09 LLESERNMDFHASNVKNLYEKLKSQLKNNAKEIGNGCFEFYHKCNDECMESVKNGTYDYP 489

***.**.:*** *********:**************************************

A/St.Louis/539/09 KYSEESKLNKEKIDGVKLESMGVYQILAIYSTVASSLVLLVSLGAISFWM 538

A/St.Louis/758/09 KYSEESKLNREKIDGVKLESMGVYQILAIYSTVASSLVLLVSLGAISFWM 538

A/St.Louis/630/09 KYSEESKLNKEKIDGVKLESMGVYQILAIYSTVASSLVLLVSLGAISFWI 538

A/St.Louis/792/09 KYSEESKLNKEKIDGVKLESMGVYQILAIYSTVASSLVLLVSLGAISFWM 538

A/St.Louis/775/09 KYSEESKLNKEKIDGVKLESMGVYQILAIYSTVASSLVLLVSLGAISFWM 538

A/St.Louis/790/09 KYSKESKLNKEKIDGVKLESMGVYQILAIYSTVASSLVLLVSLGAISFWI 538

A/St.Louis/764/09 KYSEESKLNKEKIDGVKLESMGVYQILAIYSTVASSLVLLVSLGAISFWM 538

A/St.Louis/746/09 KYSEESKLNKEKIDGVKLESMGVYQILAIYSTVASSLVLLVSLGAISFWM 538

A/St.Louis/690/09 KYSEESKLNKEKIDGVKLESMGVYQILAIYSTVASSLVLLVSLGAISFWM 538

***:*****:***************************************:
