# Supplementary material for: Inhibition of Neuraminidase Inhibitor-Resistant Influenza Virus by DAS181, a Novel Sialidase Fusion Protein
Source: PLoS One. 2009 Nov 6;4(11):e7838. doi: 10.1371/journal.pone.0007838 (PMC2770896; doi:10.1371/journal.pone.0007838)
Supplement: Figure S6 — HA alignment of recently published H1N1 IFV isolates. Published HA sequences for several 2007/2008 isolates were aligned with Clustal W2 software to determine the conservation of amino acid choice at select regions identified as mutations in Figure S3. Sequence data noted with H3 numbering scheme, as previously described [42]. Highlighted residues correspond to: Red = N163, Green = G/V189, Blue = D225. * = identical amino acid, : = highly similar amino acid, . = moderately similar amino acid. Accession numbers for HA sequences aligned here: A/Kentucky/UR06-0369/2007 = CY037663 A/Texas/UR06-0422/2007 = CY037439 A/Ohio/UR06-0493/2007 = CY037655 A/NewJersey/15/2007 = EU516083 A/Perth/33/2008 = FJ743473 A/Kentucky/UR07-0061/2008 = CY037695 A/Washington/AF06/2007 = CY037327 A/Florida/UR07-0022/2008 = CY037679 A/Hawaii/21/2007 = EU516080 A/Japan/AF07/2008 = CY037335 A/Cambodia/21/2007 = FJ743471 A/Tennessee/UR06-0106/2007 = CY037783 A/Vermont/UR06-0513/2007 = CY037463 A/Charlottesville/31/95 = AF398878 (0.05 MB DOC) [file pone.0007838.s007.doc]

A/Kentucky/UR06-0369/2007 MKVKLLVLLCTFTATYADTICIGYHANNSTDTVDTVLEKNVTVTHSVNLL 43

A/Texas/UR06-0422/2007 MKVKLLVLLCTFTATYADTICIGYHANNSTDTVDTVLEKNVTVTHSVNLL 43

A/Ohio/UR06-0493/2007 MKVKLLILLCTFTATYADTICIGYHANNSTDTVDTVLEKNVTVTHSVNLL 43

A/NewJersey/15/2007 MKVKLLVLLCTFTATYADTICIGYHANNSTDTVDTVLEKNVTVTHSVNLL 43

A/Perth/33/2008 MKVKLLVLLCTFTATYADTICIGYHANNSTDTVDTVLEKNVTVTHSVNLL 43

A/Kentucky/UR07-0061/2008 MKVKLLVLLCTFTATYADTICIGYHANNSTDTVDTVLEKNVTVTHSVNLL 43

A/Washington/AF06/2007 MKVKLLVLLCTFTATYADTICIGYHANNSTDTVDTVLEKNVTVTHSVNLL 43

A/Florida/UR07-0022/2008 MKVKLLVLLCTFTATYADTICIGYHANNSTDTVDTVLEKNVTVTHSVNLL 43

A/Hawaii/21/2007 MKVKLLVLLCTFTATYADTICIGYHANNSTDTVDTVLEKNVTVTHSVNLL 43

A/Japan/AF07/2008 MKAKLLVLLCTFTATYADTICIGYHANNSTDTVDTVLEKNVTVTHSVNLL 43

A/Cambodia/21/2007 MKVKLLVLLCTFTATYADTICIGYHANNSTDTVDTVLEKNVTVTHSVNLL 43

A/Tennessee/UR06-0106/2007 MKVKLLVLLCTFTATYADTICIGYHANNSTDTVDTVLEKNVTVTHSVNLL 43

A/Vermont/UR06-0513/2007 MKVKLLVLLCTFTATYADTICIGYHANNSTDTVDTVLEKNVTVTHSVNLL 43

A/Charlottesville/31/95 -----LVLLCAFTATYADTICIGYHANNSTDTVDTVLEKNVTVTHSVNLL 43

*:***:***************************************

A/Kentucky/UR06-0369/2007 EDSHNGKLCLLKGIAPLQLGNCSVAGWILGNPECELLISKESWSYIVETP 91

A/Texas/UR06-0422/2007 EDSHNGKLCLLKGIAPLQLGNCSVAGWILGNPECELLISKESWSYIVETP 91

A/Ohio/UR06-0493/2007 EDSHNGKLCLLKGIAPLQLGNCSVAGWILGNPECELLISKESWSYIVETP 91

A/NewJersey/15/2007 ENSHNGKLCLLKGIAPLQLGNCSVAGWILGNPECELLISKESWSYIVEKP 91

A/Perth/33/2008 ENSHNGKLCLLKGIAPLQLGNCSVAGWILGNPECELLISKESWSYIVEKP 91

A/Kentucky/UR07-0061/2008 ENSHNGKLCLLKGIAPLQLGNCSVAGWILGNPECELLISKESWSYIVEKP 91

A/Washington/AF06/2007 ENSHNGKLCLLKGIAPLQLGNCSVAGWILGNPECELLISKESWSYIVEKP 91

A/Florida/UR07-0022/2008 ENSHNGKLCLLKGIAPLQLGNCSVAGWILGNPECELLISKESWSYIVEKP 91

A/Hawaii/21/2007 ENSHNGKLCLLKGIAPLQLGNCSVAGWILGNPECELLISKESWSYIVEKP 91

A/Japan/AF07/2008 ENSHNGKLCLLKGIAPLQLGNCSVAGWILGNPECELLISKESWSYIVEKP 91

A/Cambodia/21/2007 EDNHNGKLCLLKGIAPLQLGNCSVAGWILGNPECELLISKESWSYIVERP 91

A/Tennessee/UR06-0106/2007 EDRHNGKLCLLKGIAPLQLGNCSVAGWILGNPECELLISKESWSYIVETP 91

A/Vermont/UR06-0513/2007 EDSHNGKLCLLKGIAPLQLGNCSVAGWILGNPECELLISKESWSYIVETP 91

A/Charlottesville/31/95 EDSHNGKLCRLKGTAPLQLGNCSVAGWILGNPECESLFSKESWSYIAETP 91

*: ****** *** ********************* *:********.* *

A/Kentucky/UR06-0369/2007 NPENGTCYPGYFADYEELREQLSSVSSFERFEIFPKESSWPNHTVT-GVS 136

A/Texas/UR06-0422/2007 NPENGTCYPGYFADYEELREQLSSVSSFERFEIFPKESSWPNHTVT-GVS 136

A/Ohio/UR06-0493/2007 NPENGTCYPGYFADYEELREQLSSVSSFERFEIFPKESSWPNHTVT-GVS 136

A/NewJersey/15/2007 NPENGTCYPGHFADYEELREQLSSVSSFERFEIFPKESSWPNHTVT-GVS 136

A/Perth/33/2008 NPENGTCYPGHFADYEELREQLSSVSSFERFEIFPKESSWPNHTVT-GVS 136

A/Kentucky/UR07-0061/2008 NPENGTCYPGHFADYEELREQLSSVSSFERFEIFPKESSWPNHTVT-GVS 136

A/Washington/AF06/2007 NPENGTCYPGHFADYEELREQLSSVSSFERFEIFPKESSWPNHTVT-GVS 136

A/Florida/UR07-0022/2008 NPENGTCYPGHFADYEELREQLSSVSSFERFEIFPKESSWPNHTVT-GVS 136

A/Hawaii/21/2007 NPENGTCYPGHFADYEELREQLSSVSSFERFEIFPKESSWPNHTVT-GVS 136

A/Japan/AF07/2008 NPENGTCYPGHFADYEELREQLSSVSSFERFEIFPKESSWPNHTVT-GVS 136

A/Cambodia/21/2007 NPENGTCYPGHFADYEELREQLSSVSSFERFEIFPKESSWPNHTVT-GVS 136

A/Tennessee/UR06-0106/2007 NPENGTCYPGYFADYEELREQLSSVSSFERFEIFPKESSWPNHTVT-GVS 136

A/Vermont/UR06-0513/2007 NPENGTCYPGNFADYEELREQLSSVSSFERFEIFPKESSWPNHTVT-GVS 136

A/Charlottesville/31/95 NPENGTCYPGYFADYEELREQLSSVSSFERFEIFPKESSWPNHTVTKGVT 136

********** *********************************** **:

A/Kentucky/UR06-0369/2007 ASCSHNGKSSFYRNLLWLTGKNGLYPNLSKSYANNKEKEVLVLWGVHHPP 186

A/Texas/UR06-0422/2007 ASCSHNGKSSFYRNLLWLTGKNGLYPNLSESYANNKEKEVLVLWGVHHPP 186

A/Ohio/UR06-0493/2007 ASCSHNGKSSFYRNLLWLTGKNGLYPNLSKSYANNKEKEVLVLWGVHHPS 186

A/NewJersey/15/2007 ASCSHNGESSFYRNLLWLTGKNGLYPNLSKSYANNKEKEVLVLWGVHHPP 186

A/Perth/33/2008 ASCSHNGESSFYRNLLWLTGKNGLYPNLSKSYANNKEKEVLVLWGVHHPP 186

A/Kentucky/UR07-0061/2008 ASCSHNGESSFYRNLLWLTGKNGLYPNLSKSYANNKEKEVLVLWGVHHPP 186

A/Washington/AF06/2007 ASCSHNGESSFYRNLLWLTGKNGLYPNLSKSYANNKEKEVLVLWGVHHPP 186

A/Florida/UR07-0022/2008 ASCSHNGESSFYRNLLWLTGKNGLYPNLSKSYANNKEKEVLVLWGVHHPP 186

A/Hawaii/21/2007 ASCSHNGESSFYRNLLWLTGKNGLYP**K**LSKSYANNKEKEVLVLWGVHHPP 186

A/Japan/AF07/2008 ASCSHNGESSFYRNLLWLTGKNGLYPNLSKSYANNKEKEVLVLWGVHHPP 186

A/Cambodia/21/2007 ASCSHNGESSFYKNLLWLTGKNGLYPNLSKSYANNKEKEVLVLWGVHHPP 186

A/Tennessee/UR06-0106/2007 ASCSHNGRSSFYRNLLWLTGKNGLYPNLSRSYANNKEKEVLVLWGVHHPP 186

A/Vermont/UR06-0513/2007 ASCSHNGKSSFYRNLLWLTGKNGLYPNLSKSYANNKEKEVLVLWGVHHPP 186

A/Charlottesville/31/95 ASCSHNGKSSFYKNLLWLTEKNGLYPNLSKSYVNNKEKEVLVLWGVHHPS 186

*******.****:****** ****** **.**.****************.

A/Kentucky/UR06-0369/2007 NIGDQRALYHTENAYVSVVSSHYSRRFTPEIAKRPKVRDQEGRINYYWTL 236

A/Texas/UR06-0422/2007 NIGDQRALYHTENAYVSVVSSHYSRRFTPEIAKRPKVRDQEGRINYYWTL 236

A/Ohio/UR06-0493/2007 NIGDQRALYHTENAYVSVVSSHYSRRFTPEIAKRPKVRDQEGRINYYWTL 236

A/NewJersey/15/2007 NIGNQKTLYHTENAYVSVVSSHYSRKFTPEIAKRPKVRDQEGRINYYWTL 236

A/Perth/33/2008 SISDQKTLYHTENAYVSVVSSHYSRKFTPEIVKRPKVRDQEGRINYYWTL 236

A/Kentucky/UR07-0061/2008 NIGDQKALYHTENAYVSVVSSHYSRKFTPEIAKRPKVRDQEGRINYYWTL 236

A/Washington/AF06/2007 NIGDQKALYHTENAYVSVVSSHYSRKFTPEIAKRPKVRDQEGRINYYWTL 236

A/Florida/UR07-0022/2008 NIGDQKALYHTENAYVSVVSSHYSRKFTPEIAKRPKVRDQEGRINYYWTL 236

A/Hawaii/21/2007 NIGDQKALYHTENAYVSVVSSHYSRKFTPEIAKRPKIRDQEGRINYYWTL 236

A/Japan/AF07/2008 NIGDQKALYHTENAYVSVVSSHYSRKFTPEIAKRPKVRDQEGRINYYWTL 236

A/Cambodia/21/2007 NIGDQMTLYHKENAYVSVVSSHYSRKFTPEIAKRPKVRDQEGRINYYWTL 236

A/Tennessee/UR06-0106/2007 NIGDQRALYHTENAYVSVVSSHYSRRFTPEIAKRPKVRDQEGRINYYWTL 236

A/Vermont/UR06-0513/2007 NIGDQRALYHTENAYVSVVSSHYSRRFTPEIAKRPKVRDQEGRINYYWTL 236

A/Charlottesville/31/95 NIGDQRAIYHTENAYVSVVSSHYSRRFTPEIAKRPKVRDQEGRINYYWTL 236

.*.:* ::**.**************:*****.****:*************

A/Kentucky/UR06-0369/2007 LEPGDTIIFEANGNLIAPRFAFALSRGFGSGIITSNAPMDECDAKCQTPQ 285

A/Texas/UR06-0422/2007 LEPGDTIIFEANGNLIAPRFAFALSRGFGSGIITSNAPMDECDAKCQTPQ 285

A/Ohio/UR06-0493/2007 LEPGDTIIFEANGNLIAPRFAFALSRGFGSGIITSNAPMDECDAKCQTPQ 285

A/NewJersey/15/2007 LEPGDTIIFEANGNLIAPRYAFALSRGFGSGIINSNAPMDKCDAKCQTPQ 285

A/Perth/33/2008 LEPGDTIIFEANGNLIAPRYAFALSRGFGSGIINSNAPMDKCDAKCQTPQ 285

A/Kentucky/UR07-0061/2008 LEPGDTIIFEANGNLIAPRYAFALSRGFGSGIINSNAPMDKCDAKCQTPQ 285

A/Washington/AF06/2007 LEPGDTIIFEANGNLIAPRYAFALSRGFGSGIINSNAPMDKCDAKCQTPQ 285

A/Florida/UR07-0022/2008 LEPGDTIIFEANGNLIAPRYAFALSRGFGSGIINSNAPMDKCDAKCQTPQ 285

A/Hawaii/21/2007 LEPGDTIIFEANGNLIAPRYAFALSRGFGSGIINSNAPMDKCDAKCQTPQ 285

A/Japan/AF07/2008 LEPGDTIIFEANGNLIAPRYAFALSRGLGSGIINSNAPMDKCDAKCQTPQ 285

A/Cambodia/21/2007 LEPGDTIIFEANGNLIAPRYAFALSRGFGSGIINSNAPMDECDAKCQTPQ 285

A/Tennessee/UR06-0106/2007 LEPGDTIIFEANGNLIAPRFAFALSRGFGSGIITSNAPMDECDAKCQTPQ 285

A/Vermont/UR06-0513/2007 LEPGDTIIFEANGNLIAPRFAFALSRGFGSGIITSNAPMDECDAKCQTPQ 285

A/Charlottesville/31/95 LEPGDTIIFEANGNLIAPWYAFALSRGFGSGIITSNASMGECDAKCQTPQ 285

****************** :*******:*****.***.*.:*********

A/Kentucky/UR06-0369/2007 GAINSSLPFQNVHPVTIGECPKYVRSTKLRMVTGLRNIPSIQSRGLFGAI 335

A/Texas/UR06-0422/2007 GAINSSLPFQNVHPVTIGECPKYVRSTKLRMVTGLRNIPSIQSRGLFGAI 335

A/Ohio/UR06-0493/2007 GAINSSLPFQNVHPVTIGECPKYVRSTKLRMVTGLRNIPSIQSRGLFGAI 335

A/NewJersey/15/2007 GAINSSLPFQNVHPVTIGECPKYVRSAKLRMVTGLRNIPSIQSRGLFGAI 335

A/Perth/33/2008 GAINSSLPFQNVHPVTIGECPKYVRSAKLRMVTGLRNIPSIQSRGLFGAI 335

A/Kentucky/UR07-0061/2008 GAINSSLPFQNVHPVTIGECPKYVRSAKLRMVTGLRNIPSIQSRGLFGAI 335

A/Washington/AF06/2007 GAINSSLPFQNVHPVTIGECPKYVRSAKLRMVTGLRNIPSIQSRGLFGAI 335

A/Florida/UR07-0022/2008 GAINSSLPFQNVHPVTIGECPKYVRSAKLRMVTGLRNIPSIQSRGLFGAI 335

A/Hawaii/21/2007 GAINSSLPFQNVHPVTIGECPKYVRSAKLRMVTGLRNIPSIQSRGLFGAI 335

A/Japan/AF07/2008 GAINSSLPFQNVHPVTIGECPKYVRSAKLRMVTGLRNIPSIQSRGLFGAI 335

A/Cambodia/21/2007 GAINSSLPFQNVHPITIGECPKYVRSAKLRMVTGLRNIPSIQSRGLFGAI 335

A/Tennessee/UR06-0106/2007 GAINSSLPFQNVHPVTIGECPKYVRSAKLRMVTGLRNIPSIQSRGLFGAI 335

A/Vermont/UR06-0513/2007 GAINSSLPFQNVHPVTIGECPKYVRSAKLRMVTGLRNIPSIQSRGLFGAI 335

A/Charlottesville/31/95 GAINSSLPFQNVHPVTIGECPKYVRSTKLRMVTGLRNIPSIQSRGLFGAI 335

**************:***********:***********************

A/Kentucky/UR06-0369/2007 AGFIEGGWTGMVDGWYGYHHQNEQGSGYAADQKSTQNAINGITNKVNSVI 385

A/Texas/UR06-0422/2007 AGFIEGGWTGMVDGWYGYHHQNEQGSGYAADQKSTQNAINGITNKVNSVI 385

A/Ohio/UR06-0493/2007 AGFIEGGWTGMVDGWYGYHHQNEQGSGYAADQKSTQNAINGITNKVNSVI 385

A/NewJersey/15/2007 AGFIEGGWTGMVDGWYGYHHQNEQGSGYAADQKSTQNAINGITNKVNSVI 385

A/Perth/33/2008 AGFIEGGWTGMVDGWYGYHHQNEQGSGYAADQKSTQNAINGITNKVNSVI 385

A/Kentucky/UR07-0061/2008 AGFIEGGWTGMVDGWYGYHHQNEQGSGYAADQKSTQNAINGITNKVNSVI 385

A/Washington/AF06/2007 AGFIEGGWTGMVDGWYGYHHQNEQGSGYAADQKSTQNAINGITNKVNSVI 385

A/Florida/UR07-0022/2008 AGFIEGGWTGMVDGWYGYHHQNEQGSGYAADQKSTQNAINGITNKVNSVI 385

A/Hawaii/21/2007 AGFIEGGWTGMVDGWYGYHHQNEQGSGYAADQKSTQNAINGITNKVNSVI 385

A/Japan/AF07/2008 AGFIEGGWTGMVDGWYGYHHQNEQGSGYAADQKSTQNAINGITNKVNSVI 385

A/Cambodia/21/2007 AGFIEGGWTGMVDGWYGYHHQNEQGSGYAADQKSTQNAINGITNKVNSVI 385

A/Tennessee/UR06-0106/2007 AGFIEGGWTGMVDGWYGYHHQNEQGSGYAADQKSTQNAINGITNKVNSVI 385

A/Vermont/UR06-0513/2007 AGFIEGGWTGMVDGWYGYHHQNEQGSGYAADQKSTQNAIDGITNKVNSVI 385

A/Charlottesville/31/95 AGFIEGGWTGMIDGWYGYHHQNEQGSGYAADQKSTQNAIDGITNKVNSVI 385

***********:***************************:**********

A/Kentucky/UR06-0369/2007 EKMNTQFTAVGKEFNKLERRMENLNKKVDDGFLDIWTYNAELLVLLENER 435

A/Texas/UR06-0422/2007 EKMNTQFTAVGKEFNKLERRMENLNKKVDDGFLDIWTYNAELLVLLENER 435

A/Ohio/UR06-0493/2007 EKMNTQFTAVGKEFNKLERRMENLNKKVDDGFLDIWTYNAELLVLLENER 435

A/NewJersey/15/2007 EKMNTQFTAVGKEFNKLERRMENLNKKVDDGFIDIWTYNAELLVLLENER 435

A/Perth/33/2008 EKMNTQFTAVGKEFNKLERRMENLNKKVDDGFIDIWTYNAELLVLLENER 435

A/Kentucky/UR07-0061/2008 EKMNTQFTAVGKEFNKLERRMENLNKKVDDGFIDIWTYNAELLVLLENER 435

A/Washington/AF06/2007 EKMNTQFTAVGKEFNKLERRMENLNKKVDDGFIDIWTYNAELLVLLENER 435

A/Florida/UR07-0022/2008 EKMNTQFTAVGKEFNKLERRMENLNKKVDDGFIDIWTYNAELLVLLENER 435

A/Hawaii/21/2007 EKMNTQFTAVGKEFNKLERRMENLNKKVDDGFIDIWTYNAELLVLLENER 435

A/Japan/AF07/2008 EKMNTQFTAVGKEFNKLERRMENLNKKVDDGFIDIWTYNAELLVLLENER 435

A/Cambodia/21/2007 EKMNTQFTAVGKEFNKLERRMENLNKKVDDGFIDVWTYNAELLVLLENER 435

A/Tennessee/UR06-0106/2007 EKMNTQFTAVGKEFNKLERRMENLNKKVDDGFLDIWTYNAELLVLLENER 435

A/Vermont/UR06-0513/2007 EKMNTQFTAVGKEFNKLERRMENLNKKVDDGFLDIWTYNAELLVLLENER 435

A/Charlottesville/31/95 EKMNTQFTAVGKEFNKLERRMENLNKKVDDGFLDIWTYNAELLVLLENER 435

********************************:*:***************

A/Kentucky/UR06-0369/2007 TLDFHDSNVKNLYEKVKSQLKNNAKEIGNGCFEFYHKCNDECMESVKNGT 485

A/Texas/UR06-0422/2007 TLDFHDSNVKNLYEKVKSQLKNNAKEIGNGCFEFYHKCNDECMESVKNGT 485

A/Ohio/UR06-0493/2007 TLDFHDSNVKNLYEKVKSQLKNNAKEIGNGCFEFYHKCNDECMESVKNGT 485

A/NewJersey/15/2007 TLDFHESNVKNLYEKVKSQLKNNAKEIGNGCFEFYHKCNDECMESVKNGT 485

A/Perth/33/2008 TLDFHDSNVKNLYEKVKSQLKNNAKEIGNGCFEFYHKCNDECMESVKNGT 485

A/Kentucky/UR07-0061/2008 TLDFHDSNVKNLYEKVKSQLKNNAKEIGNGCFEFYHKCNDECMESVKNGT 485

A/Washington/AF06/2007 TLDFHDSNVKNLYEKVKSQLKNNAKEIGNGCFEFYHKCNDECMESVKNGT 485

A/Florida/UR07-0022/2008 TLDFHDSNVKNLYEKVKSQLKNNAKEIGNGCFEFYHKCNDECMESVKNGT 485

A/Hawaii/21/2007 TLDFHDSNVKNLYEKVKSQLKNNAKEIGNGCFEFYHKCNDECMESVKNGT 485

A/Japan/AF07/2008 TLDFHDSNVKNLYEKVKSQLKNNAKEIGNGCFEFYHKCNDECMESVKNGT 485

A/Cambodia/21/2007 TLDFHDSNVKNLYEKVKNQLKNNAKEIGNGCFEFYHKCNDECMESVKNGT 485

A/Tennessee/UR06-0106/2007 TLDFHDSNVKNLYEKVKSQLKNNAKEIGNGCFEFYHKCNDECMESVKNGT 485

A/Vermont/UR06-0513/2007 TLDFHDSNVKNLYEKVKSQLKNNAKEIGNGCFEFYHKCNDECMESVKNGT 485

A/Charlottesville/31/95 TLDFHDSNVKNLYEKVKNQLKNNAKEIGNGCFEFYHKCNNECMESVKNGT 485

*****:***********.*********************:**********

A/Kentucky/UR06-0369/2007 YDYPKYSEESKLNREKIDGVKLESMGVYQILAIYSTVASSLVLLVSLGAI 534

A/Texas/UR06-0422/2007 YDYPKYSEESKLNREKIDGVKLESMGVYQILAIYSTVASSLVLLVSLGAI 534

A/Ohio/UR06-0493/2007 YDYPKYSEESKLNREKIDGVKLESMGVYQILAIYSTVASSLVLLVSLGAI 534

A/NewJersey/15/2007 YDYPKYSEESKLNREKIDGVKLESMEVYQILAIYSTVASSLVLLVSLGAI 534

A/Perth/33/2008 YDYPKYSEESKLNREKIDGVKLESMGVYQILAIYSTVASSLVLLVSLGAI 534

A/Kentucky/UR07-0061/2008 YDYPKYSEESKLNREKIDGVKLESMGVYQILAIYSTVASSLVLLVSLGAI 534

A/Washington/AF06/2007 YDYPKYSEESKLNREKIDGVKLESMGVYQILAIYSTVASSLVLLVSLGAI 534

A/Florida/UR07-0022/2008 YDYPKYSEESKLNREKIDGVKLESMGVYQILAIYSTVASSLVLLVSLGAI 534

A/Hawaii/21/2007 YDYPKYSEESKLNREKIDGVKLESMGVYQILAIYSTVASSLVLLVSLGAI 534

A/Japan/AF07/2008 YDYPKYSEESKLNREKIDGVKLESMGVYQILAIYSTVASSLVLLVSLGAI 534

A/Cambodia/21/2007 YDYPKYSEESKLSREKIDGVKLESMGVYQILAIYSTVASSLVLLVSLGAI 534

A/Tennessee/UR06-0106/2007 YDYPKYSEESKLNREKIDGVKLESMGVYQILAIYSTVASSLVLLVSLGAI 534

A/Vermont/UR06-0513/2007 YDYPKYSEESKLNREKIDGVKLESMGVYQILAIYSTVASSLVLLVSLGAI 534

A/Charlottesville/31/95 YDYPKYSEESKLNREKIDGVKLESMGVYQILAIYSTVASSLVLLVSLGAI 534

************.************ ************************

A/Kentucky/UR06-0369/2007 SFWMCSNGSLQCRICI 550

A/Texas/UR06-0422/2007 SFWMCSNGSLQCRICI 550

A/Ohio/UR06-0493/2007 SFWMCSNGSLQCRICI 550

A/NewJersey/15/2007 SFWMCSNGSLQCRICI 550

A/Perth/33/2008 SFWMCSNGSLQCRICI 550

A/Kentucky/UR07-0061/2008 SFWMCSNGSLQCRICI 550

A/Washington/AF06/2007 SFWMCSNGSLQCRICI 550

A/Florida/UR07-0022/2008 SFWMCSNGSLQCRICI 550

A/Hawaii/21/2007 SFWMCSNGSLQCRICI 550

A/Japan/AF07/2008 SFWMCSNGSLQCRICI 550

A/Cambodia/21/2007 SFWMCSNGSLQCRICI 550

A/Tennessee/UR06-0106/2007 SFWMCSNGSLQCRICI 550

A/Vermont/UR06-0513/2007 SFWMCSNGSLQCRICI 550

A/Charlottesville/31/95 SFWMCSNGSLQ----- 550

***********
